# A Mechanistic Evaluation of Antioxidant Nutraceuticals on Their Potential against Age-Associated Neurodegenerative Diseases

**DOI:** 10.3390/antiox9101019

**Published:** 2020-10-20

**Authors:** Nur Zuliani Ramli, Mohamad Fairuz Yahaya, Ikuo Tooyama, Hanafi Ahmad Damanhuri

**Affiliations:** 1 Department of Biochemistry, Faculty of Medicine, UKM Medical Centre, Universiti Kebangsaan Malaysia, Cheras, Kuala Lumpur 56000, Malaysia; nurzuliani@ums.edu.my; 2 Department of Biomedical Sciences and Therapeutics, Faculty of Medicine and Health Sciences, Universiti Malaysia Sabah, Kota Kinabalu 88400, Sabah, Malaysia; 3 Department of Anatomy, Faculty of Medicine, UKM Medical Centre, Universiti Kebangsaan Malaysia, Cheras, Kuala Lumpur 56000, Malaysia; mfairuzy@ukm.edu.my; 4 Molecular Neuroscience Research Centre, Shiga University of Medical Sciences, Seta Tsukinowacho, Otsu 520-2192, Shiga, Japan; kinchan@belle.shiga-med.ac.jp

**Keywords:** oxidative stress, free radical scavenger, lipid peroxidation, metal chelator, antioxidant enzymes, inflammation, cell survival, apoptosis

## Abstract

Nutraceuticals have been extensively studied worldwide due to its neuroprotective effects in in vivo and in vitro studies, attributed by the antioxidative properties. Alzheimer (AD) and Parkinson disease (PD) are the two main neurodegenerative disorders that are discussed in this review. Both AD and PD share the similar involvement of oxidative stress in their pathophysiology. Nutraceuticals exert their antioxidative effects via direct scavenging of free radicals, prevent damage to biomolecules, indirectly stimulate the endogenous antioxidative enzymes and gene expressions, inhibit activation of pro-oxidant enzymes, and chelate metals. In addition, nutraceuticals can act as modulators of pro-survival, pro-apoptotic, and inflammatory signaling pathways. They have been shown to be effective particularly in preclinical stages, due to their multiple mechanisms of action in attenuating oxidative stress underlying AD and PD. Natural antioxidants from food sources and natural products such as resveratrol, curcumin, green tea polyphenols, and vitamin E are promising therapeutic agents in oxidative stress-mediated neurodegenerative disease as they have fewer adverse effects, more tolerable, cheaper, and sustainable for long term consumption.

## 1. Introduction

Neurodegenerative disease is an irreversible condition where the function of neurons declines over time that leads to neuronal death. The incidence rates increase every year, especially in countries with an ageing population. Common neurodegenerative diseases include Alzheimer (AD) and Parkinson (PD) diseases. Worldwide, there are approximately 50 million people living with dementia, with 60–70% diagnosed with AD [1]. In America, 5.8 million individuals over the age of 65 has AD, and this is projected to increase by 6.7% between 2020 and 2025 [2]. PD is the second most widespread neurodegenerative disease after AD, and affects movement. The prevalence of PD is approximately 2% of the population older than 65 years old, involving 6.1 million people in 2016 globally [3]. Although AD and PD affect different areas in the brain and display distinctive clinical features, they share a strikingly similar pathophysiology mediated by oxidative stress.

### 1.1. Alzheimer and Parkinson Disease

AD is a brain disorder characterized by a progressive and irreversible decline in cognitive function attributed to the anomalous accumulation of neurofibrillary tangles and amyloid plaques in the brain [1]. Extracellular senile plaques (SP) and neurofibrillary tangles (NFT) are pathological hallmarks of AD. SP is generated by the fibrils of the beta-amyloid peptide (Aβ) from the proteolysis of amyloid precursor protein (APP), a transmembrane glycoprotein, by the β- and γ-secretase [4,5]. Neurofibrillary tangles (NFT) can be seen in neuron cells, which are composed of paired helical filaments (PHF) that self-aggregate due to hyperphosphorylation of tau proteins [5,6]. Recent evidence indicate that amyloid depositions occur 15–20 years before the onset of dementia, after which tau pathology develops [7,8,9]. Nevertheless, the molecular mechanism that drives the accumulation of these protein aggregates remains poorly understood, especially in sporadic cases, which hinders us from finding the right therapy for AD. Numerous evidence has shown that there is a substantial elevation of oxidative stress in AD brains, which is believed to be fundamental in the development of the disease [10]. Furthermore, Aβ oligomers, which are the precursor to amyloid fibril, display strong neurotoxicity, and the neurotoxicity is mainly due to oxidative stress induced by toxic conformer of amyloid oligomers in addition to other neurotoxic damage such as neuronal membrane disruption, microglia and astrocyte activation, as well as Ca^2+^ dyshomeostasis [11,12]. Using multiphoton imaging, researchers observed a direct association between free radical production and the existence of amyloid plaques both in AD mouse models and in human AD brain tissues, where fluorogenic free radical indicators was significantly reduced after administration of synthetic antioxidant (287.2 ± 145.6 vs. 163.5 ± 104.3, *p* < 0.001) [13]. Indeed, AD brains display elevated rates of oxidative damage, including protein, deoxyribonucleic acid (DNA), and lipid oxidation, along with the presence of redox-active metals [14].

Parkinson disease (PD), on the other hand, is clinically characterized by four cardinal motor symptoms, including bradykinesia, rigidity, resting tremor, as well as postural and gait difficulty [15]. In PD brains, there is a selective dopaminergic neuronal loss located at the substantia nigra pars compacta, and to a lesser extent in the globus pallidus, putamen, and caudate nucleus. As a result of degenerated neurons in the nigrostriatal pathway, the neurotransmitter dopamine release is reduced [16]. In the neurons of PD, aggregates of abnormal proteins are identified as Lewy bodies. They are parts of the α-Synuclein (α-Syn) protein, which is abundant in the nervous system, but has poorly understood functions [17]. α-Syn fibrillation forms aggregates that will occupy a large space in the neuron and will eventually cause neuronal death [18]. Similar to AD, oxidative stress plays a central role in the pathophysiological mechanisms underlying PD [19]. The presence of oxidized lipids, proteins, and DNA in substantia nigra of PD patients provide evidence of oxidative stress involvement [20,21]. Furthermore, dopamine metabolism by monoamine oxidase (MAO) produces hydrogen peroxide, while auto-oxidation of dopamine generates superoxide anion and reactive quinones. These reactive molecules exert cytotoxicity not only in dopaminergic neurons, but also other surrounding neurons. [22].

The debilitating effects of neurodegenerative disease warrant the search for therapeutic strategies that could delay the disease progression, restore neuronal function, and reduce neuronal death. However, there is still no definitive treatment for neurodegenerative diseases, while the treatment mainly focuses on improving the symptoms. Therefore, the potential benefits of nutraceutical compounds as neuroprotective agents against oxidative stress in neurodegenerative diseases are worth exploring. Nutraceuticals are defined as foods or food-based products containing natural bioactive compounds that promote good health with the ability to prevent and treat a wide variety of diseases and disorders [23]. The naturally occurring antioxidants found in food includes vitamins, alkaloids, and polyphenols (flavonoids, phenolic acid, and stilbenes). Neuroprotection of nutraceuticals is mediated by its antioxidant, anti-inflammatory, calcium antagonist, and anti-amyloidogenic properties. The potential of nutraceutical as neuroprotective agents has gained worldwide interest due to its availability, better tolerance, and fewer side effects. The antioxidative effects of nutraceuticals on neurodegenerative diseases are attributed to its ability to scavenge free radicals, prevent biomolecules (lipid, DNA, and protein) damage, inhibit free radical generating enzymes, activate internal antioxidant enzymes, chelate metal ions, and regulate signaling pathways that are important for cell survival [24].

## 2. Free Radicals and Oxidative Damage to Biomolecules

### 2.1. Free Radical Formation

The levels of free radicals or reactive species have been found to increase in age-related neurodegenerative disorders. Under normal conditions, low to moderate concentrations of reactive oxygen species (ROS) act as a defense mechanism in cell injury, innate immune response, regulate oxygen homeostasis, mediators in cell signaling including cell proliferation, differentiation, and apoptosis [25,26,27]. Nonetheless, elevated free radical formation can lead to neuropathophysiological conditions in oxidative stress. Significantly high ROS levels were reported in AD patients as well as in vitro and in vivo Alzheimer’s models [28,29,30]. In oxidative stress, there is an imbalance between oxidants and antioxidants in favor of the former that leads to cell damage [31]. The levels of pro-oxidants from the ROS, including superoxide oxygen radical (O_2_^•–^), hydrogen peroxide (H_2_O_2_), and hydroxyl radical (OH^•^), as well as reactive nitrogen species (RNS), including nitric oxide (NO) and peroxynitrite (ONOO^−^), are elevated. In contrast, the levels and activities of antioxidants such as glutathione peroxidase (GPX), superoxide dismutase (SOD), glutaredoxins, thioredoxins (TRX), catalase (CAT), vitamin E, and ascorbate are decreased [31,32]. In neurodegenerative diseases, mitochondria respiration is the most significant contributor of ROS [32,33]. To meet the high energy demand in the brain, glucose uptake by neuronal cells will undergo glycolysis, where the end-product pyruvate enters into the mitochondria [34]. Pyruvate is then metabolized through the pyruvate dehydrogenase and tricarboxylic acid (TCA) cycle and subsequently underwent oxidative phosphorylation in the electron transport chain (ETC) to produce ATP in the presence of oxygen. In normal conditions, approximately 0.2–2.0% of the electron leak out mainly from complex I, formed by nicotinamide adenine dinucleotide (NADH) dehydrogenases containing iron-sulfur centers, and complex III, which consist of cytochrome oxidase, which interacts with oxygen to produce O_2_^•–^ and H_2_O_2_ [35]. To prevent oxidative stress, intracellular antioxidant defenses play an essential role in maintaining the cell homeostasis by degrading the ROS. Mitochondria have two SOD isozymes, the manganese superoxide dismutase (MnSOD), or SOD2, in the matrix and the copper/zinc superoxide dismutase (Cu/ZnSOD), or SOD1, in the cytoplasm and intermembrane space that catalyze the dismutation of two O_2_^•−^ to form H_2_O_2_ [36]. H_2_O_2_ is then neutralized into water by GPX enzyme that couples the reaction with glutathione oxidation from reduced glutathione (GSH) to oxidized (GSSG) form. GSSG is then reduced back to GSH by the reduced nicotinamide adenine dinucleotide phosphate (NADPH)-dependent glutathione reductase (GR) [37]. The detoxification of H_2_O_2_ and O_2_^•−^ is vital to prevent the formation of highly reactive OH^•^ via the Fenton reaction in the presence of transition metals [38,39].

### 2.2. Brain Susceptibility to Oxidative Damage

The brain is highly vulnerable to oxidative stress because it uses a large amount of energy to maintain the neuronal synaptic activity, membrane potential and neurotransmitter synthesis, among other functions. Normally, it uses 20% of the total oxygen metabolism for energy production, even though the organ represents only 2% of the total body weight [40]. Furthermore, it is enriched with polyunsaturated fatty acids (PUFAs) that are particularly susceptible to lipid peroxidation [41,42]. Lipid peroxides (LPO) are highly reactive molecules that include malondialdehyde (MDA), 4-hydroxy-2-nonenal (HNE), acrolein, isoprostanes (IsoPs), and neuroprostanes (neuroPs), which are capable of disrupting proteins and DNA structures and functions [43,44,45]. Increased MDA, IsoPs, and HNE have been observed in the brain tissues of Tg2576 AD mice model and post-mortem AD brains [46,47]. HNE stimulated neuronal death by modifying the membrane ion transporter and various neuronal enzymes that impaired the neuronal Ca^2+^, glutamate, and glucose transport [48]. Apart from that, an imbalance in the homeostasis of transition metals such as copper (Cu), zinc (Zn), and iron (Fe) also contributes to oxidative stress by promoting OH^•^ production from H_2_O_2_ via Fenton reaction with further damage to proteins and DNA [49,50,51,52]. Protein modification through nitrosylation, carbonylation, disulphide bond formation, and glutathionylation that resist degradation by forming cross-linked protein aggregates have been documented in early AD stages [53,54]. Elevated protein carbonyl (PC) levels, a marker of protein damage, have been identified both in AD and PD patients [55]. Moreover, the oxidative damage on DNA results in the formation of oxidized base adduct including 8-hydroxyguanine (8-OHG), 8-hydroxyadenine (8-OHA), and 5,6-diamino-5-formamidopyrimidine in both nuclear and mitochondrial DNA of the brain of patients with mild cognitive impairment (MCI), the earliest clinical manifestation of AD [56]. Despite the high capacity of ROS generation in the brain, the organ’s defense mechanism against oxidative stress remains limited and reduced in ageing, due to low concentrations of endogenous antioxidants including CAT, GSH, GPX, and vitamin E compared to the liver [57,58,59,60,61,62]. The restricted regenerative capacity of postmitotic neuron cells has made oxidative stress more destructive to the brain, in which the lesion is permanent and accumulates over time compared to other organs [63,64].

### 2.3. Nutraceuticals as Free Radical Scavenger and Prevents Damage to Biomolecules

Nutraceuticals have been found to reduce free radicals by acting as a scavenger, hence preventing damage to lipid, protein, and DNA. There are many assays to measure the free radical scavenging activity of antioxidants. These assays measure the antioxidants ability to transfer a hydrogen atom (HAT) or donate an electron (ET) to free radicals, thus making them inactive. The commonly used assays include 1,1-diphenyl-2-picryhydrazyl (DPPH) assay, 2,2′-Azinobis (3-Ethylbenzothiazoline-6-Sulfonic Acid) (ABTS) assay, ferric ion reducing antioxidant power (FRAP) assay, total peroxyl radical-trapping antioxidant parameter (TRAP) assay, and oxygen radical absorbance capacity (ORAC) [65,66]. The nutraceuticals that carried out free radical scavenging activities will be further discussed in the following sections.

#### 2.3.1. Resveratrol

Resveratrol or 3,5,4′-trihydroxy-stilbene that can be found in a wide variety of plants, including red grapes, berries, apples, plums, peanuts, tea, and red wine, is a naturally occurring polyphenol compound belonging to stilbenes group [67,68]. There are two types of resveratrol isoforms, the *cis*- and *trans*-resveratrol, in which the latter exhibits more beneficial effects compared to the former. Treatment with 10 mg/kg body weight (BW)/day of resveratrol for two weeks in the angiotensin II-induced early AD rats showed a significant reduction of O_2_^•–^ in the nucleus tractus solitarius and hippocampus of the brain compared to untreated AD rats (Table 1) [69]. Similarly, piceatannol (trans-3,4,3′,5′-tetrahydroxystilbene), which has a structure analogous to resveratrol with an added hydroxyl group at the 3′ of the benzene ring, exert neuroprotective effects in neurodegenerative diseases. Treatment with piceatannol in Aβ-induced cytotoxicity PC 12 cells, a cell line derived from pheochromocytoma of the rat adrenal medulla [70], markedly reduced the intracellular ROS generation, hence improving the cell viability [71]. The direct antioxidant ability of *trans*-resveratrol was demonstrated in the intracerebroventricular-streptozotocin (ICV-STZ) infused rats, an animal model for sporadic Alzheimer-type dementia. In this study, the MDA levels were markedly increased following disease induction, but were significantly reduced after continuous treatment with 10 mg/kg BW/day of resveratrol for 21 days [72]. The radical scavenging ability of resveratrol is thought to be contributed mostly by the 4′-hydroxyl group, as it is the most reactive compared to the 3- and 5-hydroxyl groups [73]. Resveratrol’s ability to interact with hydroxyl and hydroperoxyl (^•^OOH) radicals by transferring a hydrogen in its phenol group can prevent further damage in oxidative stress [74].

#### 2.3.2. Grape Seed Extract

Apart from resveratrol found in grapes, the grape seed also possesses health benefits, due to its proanthocyanidin content. Proanthocyanidins have been documented to have the potential as a dietary supplement in the management of neurodegenerative diseases. It has been shown to act as an in vivo radical scavenger that reduces oxidative DNA and protein damage [75]. Oral administration of 100 mg/kg BW/day of grape seed extract for 30 days in aged rats significantly reduced the 8-OHdG and DNA protein cross-links in the spinal cord, cerebral cortex, striatum, and hippocampus compared to controls (Table 1) [76]. Similarly, the oxidative mediated protein damage in aged rats could also be mitigated with grape seed extract supplementation [77]. The antioxidant capacity of grape seed extract is attributed by the phenolic compound, which acts as an in vivo radical scavenger, hence reducing oxidative DNA and protein damage [75].

#### 2.3.3. *Ginkgo biloba* Extract

EGb 761 is a standardized herbal extract from the *Ginkgo biloba* plant that has demonstrated a neuroprotective ability. Administration of EGb 761 (100 µg/mL) to the transgenic neuronal cells secreting endogenous Aβ markedly reduced the level of ROS by 32% (*p* < 0.05) (Table 1) [29]. The EGb 761 is a potent antioxidant that can function in both aqueous solution (hydrophilic) and liposomes or a low-density lipoprotein (hydrophobic) environment [78]. Additionally, *Ginkgo biloba* extract has been shown to improve attention, memory, and cognition among human subjects in several randomized controlled trials [79,80].

#### 2.3.4. Green Tea Polyphenols

Polyphenols found in green tea could also exert a productive radical scavenging activity. Examples of the green tea polyphenols include epigallocatechin-3-gallate (EGCG), epicatechin-3-gallate (ECG), epigallocatechin (EGC), and epicatechin (EC), which belongs to the flavanols group. Each of them has a different degree of lipid and hydroxyl radical scavenging abilities that is attributed to the ortho-3′, 4′-dihydroxy moiety or an ortho-trihydroxyl group in their structures [81,82]. The neuroprotective effects of green tea have been demonstrated by Arab et al. [83]. In this interventional study, four pills amounting to 2 g of green tea/day in two divided doses were given daily for two months to 30 patients with severe AD. Each pill contained 50 mg of total polyphenols, including EGCG, EC, and ECG. At the end of the study, the total antioxidant capacity of plasma as measured by a FRAP assay was significantly improved as compared to baseline values (1140.7 ± 69.0 vs. 1391.1 ± 54.9, *p* = 0.000) (Table 1). Correspondingly, marked reduction in the levels of 8-OHdG (957.0 ± 52.5 to 719.7 ± 39.6 ng/mL, *p* = 0.001), MDA (4.3 ± 0.7 to 2.4 ± 0.3 nmol/mL, *p* < 0.005), and PC (1.9 ± 0.4 to 1.2±0.3 nmol/mg, *p* < 0.05) were observed as well [83].

#### 2.3.5. Curcumin

Across Asia, turmeric is one of the main spices used in cooking. It is also routinely used in traditional Chinese and Indian medicine. It comes from a rhizomatous plant of the ginger family, known as Zingiberaceae. Turmeric extracts include curcumin, demethoxycurcumin, and bisdemethoxycurcumin, which belong to the curcuminoid family. Curcumin, (1,7-bis(4-hydroxy-3-methoxyphenyl)-1,6-heptadiene-3,5-dione) is a turmeric polyphenol that causes yellow pigmentation. Its antioxidant capacity has been demonstrated by Frautschy et al. [84], where supplementation of 2000 parts per million (ppm) dietary curcumin for two months reduced brain IsoPs levels in the ICV infusion of Aβ peptides in rats compared to Aβ treated rats on control diet (averaged value was 15 and 45 pg/mg, respectively; *p* < 0.005) (Table 1). Curcumin treatment at 500 ppm for six months was also shown to suppress LPO in the lumbar spine of Tg2576 APP transgenic mice that resulted in rescued motor function deficits compared to untreated transgenic Tg2576 APP mice (relative intensity of anti-HNE immunoreactivity level was 3.5 and 5.0, respectively; *p* < 0.05) [85]. Several studies have reported that the phenolic hydroxyl group in the curcumin structure is responsible for the inhibition of lipid peroxidation [86,87]. While curcumin has been shown to exert beneficial antioxidative effects against neurodegenerative disease, its efficacy is limited due to poor bioavailability, solubility, and structural stability [88]. Nevertheless, modification of curcumin by conjugating it with a carrier, encapsulation within the nanoparticle, and modification of its chemical structure have rendered positive results, including enhanced activity towards cancer cells, better wound healing properties, and strong antibacterial effects against *Pseudomonas aeruginosa* and *Staphylococcus aures* [89,90]. Shelat et al. [91] reported that treatment with oral CUR-CA-THIONE, an alternative formulation derived from curcumin using glutathione and casein as vectors for better water solubility and brain bioavailability [89], at a dose of 500 mg/kg BW/day for 15 days to the aluminum chloride (AlCl_3_)-induced AD Wistar rats successfully inhibited LPO formation as measured by a thiobarbituric acid reactive substances (TBARS) assay (*p* < 0.05) compared to untreated AD rats, which was associated with improved locomotor and exploratory behavior. Moreover, in an in vitro study, a curcumin derivative, demethoxycurcumin, reduced intracellular ROS generation induced by neurotoxic rotenone [92].

#### 2.3.6. Xanthorrhizol

Javanese turmeric or *Curcuma xanthorrhiza* Roxb. is another type of turmeric from the Zingiberaceae family that is commonly located on the island of Java, Indonesia and the neighboring Southeast Asian countries. Its bioactive compound includes Xanthorrhizol, which is a sesquiterpenoid extracted from the *Curcuma xanthorrhiza* Roxb. rhizomes. Xanthorrhizol (0.5, 1, 5, and 10 µM) showed an inhibitory effect on H_2_O_2_-induced lipid peroxidation in rat brain homogenate in a dose-dependent manner, in which the highest dose reduced lipid peroxidation by 101.87 ± 1.62% of control (*p* < 0.001) [93]. Furthermore, 2 µM of Xanthorrhizol suppressed glutamate induced-ROS generation in the mouse hippocampal neuronal HT22 cells (130 vs. 210% of control, *p* < 0.05) that was comparable to that of curcumin (Table 1) [93].

#### 2.3.7. Magnolol

Traditional Chinese and Japanese medicine have been using magnolol (5,5′-diallyl-2,2′-dihydroxy biphenyl), a bioactive polyphenolic compound isolated from the stem bark of *Magnolia officinalis*, for the management of a wide variety of diseases, including gastrointestinal and respiratory disturbances. Magnolol bark extract can also be found in chewing gum as prevention against plaques and dental caries. Previously, the beneficial effects of magnolol against neurodegenerative diseases have also been demonstrated. Supplementation of 30 mg/kg BW single dose of magnolol after induction of parkinsonism using 1-methyl-4-phenyl-1,2,3,6- tetrahydropyridine (MPTP) in mice exhibited a significant reduction in the thiobarbituric acid reactive substances (TBARS) level, an indicator of lipid peroxidation, compared to rats that received a vehicle solution (110 vs. 168% of control) (*p* < 0.05) (Table 1) [94]. In another study, extracts of *Magnolia cortex* at 1 mg/mL concentration reportedly exhibited the highest inhibitory effects on lipid peroxidation as measured by TBARS assay compared to other Kampo drugs (traditional Chinese/Japanese crude drugs) (73.15% vs. 0.00–62.55%) [95]. Further identification of this extract by high-performance liquid chromatography (HPLC) revealed magnolol as one of the main phenolic constituents [95]. Likewise, the pre-treatment of magnolol (16 and 32 µM) in acrolein-induced oxidative damage in neuroblastoma cells markedly reduced the superoxide anions, LPO, and PC levels as opposed to the untreated acrolein-exposed cells [96].

#### 2.3.8. Pycnogenol^®^

Pycnogenol is a patented name for the standardized extract exclusively from the bark of French maritime pine. It contains a mixture of a variety of bioflavonoids, including catechin, epicatechin, taxifolin, procyanidins, and phenolic acids [97]. Traditionally, the pine bark was used for the treatment of wounds and scurvy and has now progressed to be an effective nutritional supplement for various chronic diseases. Pycnogenol demonstrates an effective scavenging activity against oxygen free radicals and stable radicals. The pycnogenol quenching capacity towards DPPH radicals is proportional to Trolox [98]. Pre-treatment of 100 μg/mL pycnogenol on acrolein-induced cytotoxicity in neuroblastoma cells significantly reduced ROS (190 vs. 470% of control), superoxide anions (150 vs. 260% of control), PC (140 vs. 190% of control), and 4-HNE bound proteins (130 vs. 200% of control) formation compared to untreated acrolein-exposed cells (all *p* < 0.001) (Table 1) [99]. In the hippocampus and cerebral cortex of ICV-STZ induce AD rats, pycnogenol pre-treatment at a dose of 10 mg/kg BW for three weeks remarkably reduced the PC content (PC_hippocampus_: 35 vs. 45 nmol/mg; PC_cerebral cortex_: 40 vs. 50 nmol/mg) and TBARS levels (TBARS_hippocampus_: 2.0 vs. 3.0 nmol/mg; TBARS_cerebral cortex_: 5.0 vs. 7.0 nmol/mg) in contrast to the untreated ICV-STZ rats, ultimately improved the cognitive performance (all *p* < 0.05) [100]. Antioxidant capacity of pycnogenol has also been demonstrated in a clinical trial. In this trial, 25 healthy individuals supplemented with 150 mg/day of pycnogenol for six weeks showed a 40% increase in the plasma ORAC against baseline values (3.5 vs. 2.5 µM Trolox equivalent, *p* < 0.05) [101]. Furthermore, smokers who were prescribed with 50 mg of pycnogenol had lowered plasma reactive oxygen metabolites after two weeks by 25.3% from the baseline value (459.4 ± 65 vs. 342.2 ± 56 Carr units) compared to those receiving a placebo [102].

#### 2.3.9. Guarana Seed Extract

Guarana seed is native to the South American continent, especially in the Amazon region, and are primarily consumed by the Brazilians as a dietary supplement and energy drink. It contains very high levels of caffeine as well as polyphenols such as catechins and epicatechins [103]. Recently, in vitro antioxidant assay done on a variety of Brazilian herb extracts, including from guarana seeds, have been reported [104]. Moreover, the antioxidant and anti-ageing potential of guarana seed, together with its protective effect on neurodegenerative diseases, has been well documented [105,106,107]. Guarana seed extract (100 and 1000 μg/mL) exerts significant antioxidant capacity as shown by a TRAP assay that was similar to 40 μg/mL of caffeine. In addition, there was a significant reduction of intracellular ROS production in acrolein-induced cytotoxicity on human neuronal-like cells to similar levels in control cells (*p* < 0.0001) (Table 1) [108]. Comparably, Boasquívis et al. [107] demonstrated that 5, 10, and 50 mg/mL of guarana hydroalcoholic extract (GHE), which contains 166.07 μg/mL of caffeine, 36.35 μg/mL of EC, 34.59 μg/mL of catechin, and 2.49 μg/mL of theobromine, has a similar DPPH radical scavenging property as 500 μM of Trolox, a water-soluble analogue of vitamin E. Moreover, either a dose of 10 or 50 mg/mL of GHE markedly reduced intracellular ROS levels in *C. elegans* models of AD compared to untreated control (*p* < 0.05) and eventually prolonged the worm’s lifespan [107]. The antioxidant ability of guarana seed extract is due to the synergistic effect of polyphenol compounds together with its high caffeine content [106].

#### 2.3.10. Vitamin E

Vitamin E is a lipid-soluble vitamin that is primarily found in vegetable oils such as palm, coconut, sunflower, soybean, and wheat germ oils [109]. There are two classes of vitamin E, namely tocopherols and tocotrienols. Each class has four isoforms comprising α-, β-, γ-, and δ- tocopherols as well as α-, β-, γ-, and δ- tocotrienols. They exhibited strong antioxidative properties that resulted in health-promoting effects such as in attenuating osteoporosis, cardiovascular, and neurodegenerative diseases [110,111,112,113]. The direct antioxidative properties of vitamin E include its ability to donate hydrogen atom from its chromanol ring that neutralizes free radicals [114]. In this process, tocopherol and tocotrienol become oxidized to form stable tocopheroxyl- and tocotrienoxyl-radicals. Due to its lipid solubility, vitamin E is distributed mainly in the lipid membrane, which is crucial in terminating the lipid peroxidation chain reactions. These molecules can be converted back to their reduced states by interacting with other endogenous antioxidant systems to continue the scavenging process. Supplementation of tocotrienol rich fraction (TRF) (150 mg/day) for six months to healthy older adults significantly reduced MDA (0.14 vs. 0.22 nmol/mL) and DNA damage levels (95 vs. 125 arbitrary unit, AU) compared to their baseline values (both *p* < 0.05) (Table 1) [115]. Moreover, the administration of TRF (200 mg/kg BW) for eight months to healthy Wistar rats decreased DNA damage in comparison with control group (2.87 ± 0.48 vs. 5.96 ± 0.43%, *p* < 0.05), which was associated with improved cognitive function [116]. Similar results were also observed in APPswe/PS1dE9 mice, a transgenic AD mouse model that displays elevated Aβ plaque due to Swedish mutations in APP and deleted presenilin-1 in exon 9. Significant reduction of DNA damage (3.47 ± 0.35 vs. 14.04 ± 1.475%, *p* < 0.05) following supplementation with TRF at 200 mg/kg BW daily of for six months was observed compared to the unsupplemented control mice [117].

## 3. Metal Ions and Neurodegeneration

### 3.1. Pathophysiology of Neurodegenerative Disease Involving Transition Metals

Essential transition metals, especially Cu, Zn, and Fe, are involved in many brain functions. These include oxygen carrier, redox balance, DNA synthesis, oxidative phosphorylation, myelogenesis, enzymatic activities, as well as neurotransmitter synthesis and metabolism [118]. However, their concentrations in the brain are tightly regulated to maintain the metal-ion homeostasis, as abnormally high concentrations would result in neuronal death and neurodegenerative diseases. It has been proposed that disruption in the metal-ion homeostasis is one of the key factors for the development of AD and PD [119]. A high concentration of Cu, Zn and Fe have been found in amyloid plaque [120]. Cu and Zn could bind directly to Aβ with high affinity and aggravate the fibril formation [121]. In contrast, iron is present mostly in a ferritin bound form in neuritic processes related to the amyloid plaques [122]. The Cu-Aβ complex can generate ROS in a reaction catalyzed by reducing agent such as ascorbate. Ascorbate could reduce Cu^2+^ to Cu^+^. Subsequently, the latter could donate an electron to molecular oxygen forming superoxide anion. In turn, the interaction between superoxide anion with another Cu^+^ along with additional hydrogen ions produces H_2_O_2_ [123]. Meanwhile, Cu^+^ could also reduce H_2_O_2_ to OH^•^ in a process analogous to Fenton reaction [124].

In the mitochondria, complex I and III that contains iron-sulfur (Fe-S) are susceptible to ROS attack, releasing the free ferrous iron into the mitochondrial matrix, increasing the labile iron pool [119]. The free iron can either be expressed as either Fe^2+^ or Fe^3+,^ which can facilitate the Fenton reaction and induce lipid peroxidation of the mitochondrial membrane forming mitochondrial permeability transition pore that further aggravates oxidative mitochondrial damage [125]. In dopaminergic neuronal cells, iron-mediated dopamine oxidation produces neurotoxic dopamine o-quinones and 6-hydroxydopamine (6-OHDA) [126]. Quinones which is the major by-product of dopamine oxidation can damage proteins and DNA through protein alkylation, and because quinone is a redox-active compound, it can go through redox cycling with semiquinone, generating O_2_^•−^ [127]. Besides, quinones readily react with GSH, resulting in low GSH concentration. Meanwhile, 6-OHDA can inhibit complexes I and IV (cytochrome c oxidase) of the ETC, compromising the mitochondrial function, eventually leading to cell death [128]. 6-OHDA is also oxidised by iron to reactive semiquinone, and further metabolism of 6-OHDA generates H_2_O_2_ that can be involved in another Fenton reaction [129].

### 3.2. Metal Chelation in the Treatment of Neurodegenerative Diseases

In regard to metal ions function in maintaining neurophysiology, their abnormal accumulation in the brain might promote the development of neurodegenerative diseases by creating a pro-oxidant environment. One of the practicable means of reducing oxidative stress is by targeting the transition metals using metal ion chelators [130]. In 6-OHDA-induced PD rats, intranasal treatment with iron chelator deferoxamine improved the motor activity [131], while in an in vitro study, deferoxamine attenuated iron-mediated oxidative stress in SK-N-SH, a dopaminergic cell line [132]. In a clinical trial conducted by Devos et al. [133], oral deferiprone was administered to PD patients for 12 months, led to a reduction of iron deposit in substantia nigra as quantified by magnetic resonance imaging, and improved motor function as assessed by the Unified Parkinson’s Disease Rating Scale. However, when treatment was discontinued, the positive effects were reversed [133]. Although the use of metal chelators like deferoxamine who have high affinities for Fe, aluminium, Cu, and Zn is approved in the clinical setting, it is also important to note the side effects caused by this treatment. Toxicity associated with deferoxamine includes visual and hearing loss, growth retardation, and neurotoxicity [134]. Due to the severe side effects of metal chelators, it is imperative to investigate other alternative treatment strategies using natural products that are safer, more tolerable and effective [135].

### 3.3. Nutraceutical that Acts as Metal Chelators

#### 3.3.1. Epigallocatechin Gallate (EGCG)

One of the critical steps of PD is the formation of Lewy bodies that contains aggregations of insoluble fibrils of modified α-Syn. The presence of Fe^3+^ promotes α-Syn toxic aggregation and contributes to the generation of OH^•^ [136]. Therefore, Fe^3+^ chelation is crucial in relieving the neurotoxicity associated with abnormal fibrillation. The EGCG compound found in green tea contains many health benefits, including metal chelators. In a molecular and cellular study, 20 µM EGCG inhibited the accelerated formation of α-Syn fibrillation and β-sheet conformers in Fe^3+^-induced α-Syn fibrillation. The thioflavin T (ThT) fluorescence assay and circular dichroism (CD) spectroscopy was used to measure conformational transition of α-Syn from random coil to β-sheet in this study. After addition of EGCG and 20 µM of Fe^3+^ in α-Syn, the maximum fluorescence intensity at 24 h decreased with increased lag phase against Fe^3+^-induced α-Syn fibrillation alone (0.85 vs. 1.0 fluorescence intensity, respectively) indicating a reduction in β-sheet content and delayed the formation of β-sheet conformer. Similar results was observed in CD spectroscopy, in which the negative ellipticity were reduced with EGCG treatment slowing down the formation of β-sheet conformer compared to Fe^3+^-induced α-Syn fibrillation alone at 8 h incubation time (−22.5 vs. −26 mdeg, respectively) Furthermore, EGCG at different EGCG/Fe^3+^ ratios (0.5, 1 and 2) significantly reduced the intracellular ROS generation in contrast with Fe^3+^-induced-ROS in the PC 12 cells (<0.5 vs. >1.2 relative fluorescence intensity, respectively) (Table 2) [137]. EGCG contains phenolic hydroxyls in B and D rings that react with Fe^3+^ to form a 2:1 metal-ligand complex hence prevented the interaction of Fe^3+^ with α-Syn [138]. In a similar manner, the polyphenols EGCG has also shown metal chelation ability towards divalent metal such as Cu^2+^. The EGCG interaction with Cu^2+^ inhibited the formation of α-Syn fibrillation, β-sheet conformation, and restructure amyloid fibrils into soluble amorphous aggregates in Cu^2+^-induced fibrillation of α-Syn. ThT fluorescence assay showed a reduction in fluorescence intensity when 20 µM of EGCG were added to 40 µM of Cu^2+^ in α-Syn compared to Cu^2+^-induced fibrillation in α-Syn alone (0.8 vs. 3.5 fluorescence intensity, respectively) [139]. It is also reported that the EGCG/Cu^2+^ complex bind to Tyr residue in the aromatic region of α-Syn to prevent fibrillation.

Transferrin receptor (TfR) is vital for iron metabolism, where after binding to transferrin, it internalized the transferrin iron complex and provide target cells with iron. Measuring the TfR level provides a reliable indicator of iron status [140]. This regulation is mediated by binding of trans-acting iron regulatory proteins (IRP) to iron-responsive elements (IRE) at its 3′-end of TfR messenger ribonucleic acid (mRNA), allowing for TfR synthesis in low iron concentration. In contrast, in a high concentration of iron, IRP will bind to excess iron instead, resulting in TfR mRNA degradation. Concerning this, EGCG pre-treatment at the dose of 10 µM in SH-SY5Y cells, human-derived neuroblastoma cell line as a cell model of neurodegenerative disease, significantly increased the TfR to 185 ± 20% of control and 5 µM of EGCG induced TfR mRNA expressions to 336 ± 33% of control, indicating a reduced in the intracellular iron pool due to the chelating activity of EGCG [141]. This study also compared the metal chelation ability of EGCG with desferrioxamine, and the result was insignificant. Besides, EGCG has an added benefit in reducing APP expression via activation of an (IRE-type II) in the 5′ untranslated region (UTR) of APP mRNA, hence reducing Aβ generation.

#### 3.3.2. Curcumin

Curcumin can limit oxidative stress by binding to redox-active transition metals. Pre-treatment of BNL CL.2, cell line derived from murine embryonic liver cells, with 12.5, 25 and 50 µM of curcumin decreased ferritin heavy (H) and light (L) subunit to approximately 65% of control despite increasing their mRNA expression in a dose-dependent manner as observed from the Western and Northern blot analysis (Table 2) [142]. Ferritin, an iron storing protein contains two subunits, L and H. In the event of iron-depleted states or iron-chelation therapy, IRP will bind to IRE at 5′-end of the ferritin mRNA, preventing its translation, hence reducing less iron to be stored. This discrepancy results indicate that curcumin selectively inhibits ferritin mRNA translation. In the same study, curcumin showed the potential to act as an iron chelator in vivo in which mice that were given 2% of curcumin in their dietary constituent for twelve weeks also showed similar results. The ferritin H and L expression were significantly reduced by 0.6- and 0.5-fold than control, respectively. Furthermore, in 6-OHDA-induced animal models of PD, intragastric administration of curcumin for twenty-four days, reduced the iron-positive cells count in substantia nigra using the Perls’ iron staining method compared to untreated control (25 vs. 42 number of iron-stained cells, respectively; *p* < 0.05) [143]. The strong binding of curcumin to Fe^2+^ was shown in ferrozine assay, where approximately 38.9% of Fe^2+^ chelated at the final concentration of curcumin (30 µg/mL) reduced redox cycling of iron hence inhibited Fe^2+^ and quinolinic acid-induced lipid peroxidation in rat’s brain homogenate [144]. The addition of Fe^2+^ and quinolinic acid increased the MDA levels to 0.43 and 0.38 nmol/mg, respectively, whereas curcumin treatment at 25 µM significantly inhibited this effect by reducing the MDA level to 0.15 and 0.18 nmol/mg, respectively. Quinolinic acid stimulates lipid peroxidation by forming a complex with iron, but curcumin reversed this action by binding with iron, depleting the available iron to form the pro-oxidant complex. Interestingly, because curcumin has a high affinity to metal, the curcumin-metal complex possesses antioxidant activity that is more potent than their parent compound. Yan et al. [145] demonstrated that curcumin-Cu^2+^ and curcumin-Zn^2+^ complexes significantly reduced intracellular ROS, MDA and increased CAT, SOD and GPX in H_2_O_2_-induced injury in neuronal PC 12 cells. The ratios of 2:1 curcumin-Cu^2+^, 1:1 curcumin-Cu^2+^, 2:1 curcumin-Zn^2+^, and 1:1 curcumin-Zn^2+^ complexes significantly reduced ROS levels by 150, 175, 175 and 200% of control, respectively, as well as MDA levels by >110% of controls. Furthermore, the increased in the antioxidant enzymes CAT (≥40% of control), SOD (≥90% of control) and GPX (≥60% of control) were observed. Curcumin contains two phenolic groups and a keto-enolic moiety metal-chelating sites that interact with metal ions such as Fe^2+^, Cu^2+^, and Zn^2+^ and can form 2:1 configuration or 1:1 in conjunction with other ligands [146]. Due to its lipophilic nature, curcumin can easily cross the membrane and chelate more metal ions protecting against metal toxicity in neurodegenerative diseases [147].

## 4. Dysregulation of Antioxidant and Pro-Oxidant Enzymes

Mechanisms to regulate the levels of free radicals utilize enzymatic and non-enzymatic approaches [148]. Enzymatic antioxidants include CAT, SOD, GPX, GR, thioredoxin reductase (TrxR), heme oxygenase (HO), biliverdin reductase, and peroxiredoxin [149]. Meanwhile, non-enzymatic antioxidants comprised of uric acid, GSH, melatonin, bilirubin, polyamines, and vitamins [150]. These molecules could act directly on the radicals and halt the oxidative chain reaction. Although the majority of ROS is produced as by-products of mitochondrial respiration, other oxidases enzymes, referred to as pro-oxidant enzymes, also contribute to the accumulation of ROS.

### 4.1. Internal Antioxidation Enzymes

SOD, CAT, and GPX are one of the first-line defense mechanisms against ROS. Treatment with SOD mimetic Manganese 5,10,15, 20-tetrakis (4-benzoic acid) porphyrin (MnTBAP) in SOD-deficient mice prevented the formation of liver and heart pathologies, but the progressive neuropathological lesions still remains. Since this compound could not cross the blood brain barrier, this study highlighted the importance of endogenous enzymatic antioxidant SOD in the brain [151]. Furthermore, a study by Youssef et al. [152] showed that there was a reduction of total GPX activity in the superior temporal gyrus of post-mortem AD patients compared to control where the accumulation of H_2_O_2_ occurs despite a slight compensatory increase in the GPX protein. Moreover, in PD and dementia with Lewy bodies tissue, the distribution of GPX was highly concentrated in the microglial cells surrounding the Lewy bodies, which contained α-syn that was capable of producing H_2_O_2_ [153]. The non-enzymatic reaction of GSH against ROS, such as O_2_^•−^, NO, OH^•^, and ONOO^−^, has been demonstrated in previous studies [154,155]. In PD patients, there was a 20% reduction in GSH, especially in the striatum, compared to healthy control [156]. Low levels of GSH in PD might be the result of defective synthesis, excessive metabolism, or abnormal utilization that is associated with oxidative stress in ageing [157]. Additionally, the GSH and GSH/GSSG ratio were also reduced in AD and MCI patients compared to healthy subjects [158].

### 4.2. Gene Expression for the Antioxidant Enzymes

Genetic regulations of the antioxidant defense system are under the control of nuclear factor erythroid 2-related factor 2 (Nrf2). Also known as the master regulator of the antioxidant response, Nrf2 is encoded by the nuclear factor, erythroid 2 like 2 (*NFE2L2*) gene [159]. Nrf2 is regulated and suppressed by the kelch-like ECH-associated protein 1 (Keap1)-binding protein that suppresses Nrf2 under physiologic conditions [160]. In response to redox stressed condition, ROS could modify Keap1 proteins, preventing it from binding to Nrf2 that resulted in nuclear translocation. In the nucleus, Nrf2 binds to antioxidant/electrophile response element (ARE/EpRE) sequences in the promoter region of its target genes and activates their transcription [161]. In Nrf2-deficient mice with amyloidopathy and tauopathy, there was increased oxidation and inflammation markers, along with elevated levels of phosphorylated Tau protein and Aβ*56 oligomers compared to mice with normal Nrf2 levels [162]. In a comparative post-mortem study, the expression of Nrf2 in the AD brains were mostly located in the cytoplasm of the hippocampi compared to equal nuclear and cytoplasmic Nrf2 expression in the control brains [163]. These findings indicated that the Nrf2-mediated antioxidant response was compromised in AD, although the presence of oxidative stress was evident.

### 4.3. Pro-Oxidant Enzymes

Nitric oxide synthase (NOS), xanthine oxidase (XO), and NADPH oxidase (NOX) are the main pro-oxidant enzymes in neurodegenerative diseases. NOS produces NO, a signaling molecule with multiple targets, in the presence of arginine and oxygen as its substrates [164]. To date, three isoforms of NOS have been identified, namely eNOS (endothelial NOS), nNOS (neuronal NOS), and iNOS (inducible NOS) [164]. Aβ has been shown to stimulate NOS activity in cultured astrocytes or microglia [165]. The resultant NO is susceptible to oxidation as it contains an unpaired electron that can readily react with other molecules. Interaction between NO with superoxide anion leads to the formation of peroxynitrite [164]. Peroxynitrite is a lipid permeable molecule with a highly oxidizing capacity that can cause damage to DNA, RNA, lipid, and proteins. Breakdown of peroxynitrite could also release hydroxyl radical, a more radical agent [166]. During oxidation in purine catabolism, XO interacts with oxygen, leading to O_2_^•−^ and H_2_O_2_ formation [167].

### 4.4. Modulation of Antioxidant and Pro-Oxidant Enzymes by Nutraceuticals

#### 4.4.1. Resveratrol

Resveratrol could reduce oxidative stress by stimulating the endogenous antioxidant enzymes. This was shown by Mokni et al. [168], where intraperitoneal resveratrol injection of 12.5 mg/kg BW/day for seven days in healthy Wistar rats increased the activity of antioxidant enzyme SOD (2.60 ± 0.09 U/mg), CAT (1.32 ± 0.12 nmol/min/mg), and peroxidase (19.20 ± 3.00 nmol/min/mg) in the brain compared to control (1.60 ± 0.14 U/mg, 0.48 ± 0.03 nmol/min/mg, and 9.00 ± 3.60 nmol/min/mg; respectively, *p* < 0.05) (Table 3). The result was corroborated by another study in lymphoblastoid cell lines of AD patients, whereby 50 µM of resveratrol treatment significantly upregulated the gene expression of the antioxidant system including *CAT*, *SOD2*, and *NFE2L2* compared to AD control (relative mRNA expression: 3 vs. 1, 2.5 vs. 1, and 2.5 vs. 1, respectively) [169]. Among all of these, the upregulation of *NFE2L2* by resveratrol was the most important in activating the antioxidation system as it encoded the Nrf2. Not only did 15 µM resveratrol increased Nrf2 gene expression, it also initiated Nrf2 translocation in the nucleus resulted in the increased in the mRNA and protein expression of heme oxygenase-1 (HO-1) (increased intensity on 1.0% agarose gel electrophoresis and western blot analysis, respectively), on PC 12 cells models of PD. In addition, resveratrol appeared to enhance gene expression of catalytic glutamyl-cysteine ligase (GCLC) mRNA levels as observed in the 1.0% agarose gel electrophoresis, which improved its catalytic activity as well as cell survival. The synthesis of GSH depends on the availability of substrates such as cysteine and the rate-limiting enzyme activity, glutamyl-cysteine ligase (GCL) [170]. Both HO-1 and GCLC were under the Nrf2 control, all of which were activated by resveratrol. Together, they provided robust cellular protection against oxidative stress [171].

#### 4.4.2. Curcumin

Apart from induction of parkinsonism via MPTP, the disease could also be modelled using its active metabolite MPP^+^. Similar to MPTP, direct MPP^+^ administration could result in dopaminergic neuronal death, mitochondrial damage, oxidative stress, and enhanced iNOS levels. Co-treatment with free curcumin of 20 µmol/L and MPP^+^ to PC 12 cells, significantly decreased apoptosis by attenuating the rate of iNOS overexpression (71.9 ± 4.0% to 40.0 ± 3.0%; *p* < 0.01) (Table 3). Interestingly, the neuroprotection effects of curcumin in this study were equal in comparison with aminoguanidine, an iNOS-specific inhibitor [172]. Besides downregulating pro-oxidants, curcumin has the potential to activate the phase II enzymes, which are under the control of the Nrf2-ARE pathway. The role of phase II enzymes in conjugation reactions of xenobiotic detoxification and neutralizing free radicals is an essential defense mechanism in the nervous system [173]. Curcumin incubation at 5, 10, and 15 µM significantly ameliorates oxidative stress in primary astrocytes through the activation of phase II enzymes, namely HO-1, NADPH: quinine oxidoreductase 1 (NQO1) (both HO-1 and NQO1 showed ≥200% protein levels of controls), and GCLC (>50% protein levels of controls) [174]. It appeared that phase II enzymes activation by curcumin occurred through Nrf2 stabilization and binding to the ARE hence increasing the total antioxidant capacity. In neurodegenerative disease, GSH is often exhausted, leading to oxidative stress and decreased in the tyrosine hydroxylase (TH) activity, which was the early step in dopamine synthesis. In a study using rotenone-induced PD rats, Cui et al. [175] reported decreased TH activity along with decreased HO-1, NQO1, and GSH in the substantia nigra pars compacta. These changes were successfully reversed by curcumin treatment 100 mg/kg BW twice a day for 50 days that was achieved via phosphorylation and activation of protein kinase B (Akt) and Nrf2 (increase percentage: 152%, 79.3%, and 104% for HO-1, NQO1, and GSH, respectively) [175]. Likewise, curcumin at the dose of 15, 20, and 30 µM increased HO-1 enzyme expression by 1.8, 2.0, and 2.5 vs. 1.0 AU in control in iodoacetate-induced neurotoxicity in cerebellar granule neurons and protected them from cell death [176]. In another cell culture model from C6 rat glioma cells, 5 µM of curcumin positively regulated the antioxidative mechanism by increasing the gene expression of HO-1, NQO1, CAT, TrxR, and GCLC by log ratio of (3.5, 1.5, 0.5, 1.6, and 1.0 relative to control, respectively) after 24 h of treatment [177].

#### 4.4.3. Green Tea Polyphenols

Green tea contains polyphenols that are rich in flavonols such as EGCG. EGCG had a variety of health benefits including, anti-inflammatory, anticarcinogenic, anti-angiogenic, and antioxidant effects. Apart from direct antioxidant ability, the neuroprotective effect of EGCG is also manifested indirectly through modulation of the antioxidant enzymes. Administration of EGCG at 2 mg/kg BW/day to MPTP-induced parkinsonism in male C57/BL mice, an inbred strains of mice, for 10 days significantly prevented the increased antioxidant enzymes activities of SOD and CAT by 140% and 110%, respectively, in the striatum compared to control (Table 3) [178]. In another study using a similar model, high doses of EGCG at 10 and 50 mg/kg BW/day intraperitoneally for 14 days significantly attenuated the increased iNOS expression in the substantia nigra and striatum by 100% and 102% of controls, respectively. The authors believed that EGCG posed an ability to block neuronal uptake of MPP^+^ hence prevented glial reaction and subsequent neuronal death [179]. The effects were similar when 1, 5, and 10 µM of EGCG was given to lipopolysaccharide (LPS)-induced activated micgrolia, where there was a dose-dependent reduction of iNOS levels (relative levels were 1.13, 0.75, and 0.5% when controlled with β-actin vs. 2.13% in untreated activated microglia [180]). The indirect antioxidation properties of EGCG is evident not only during a short-term treatment but also in the long term. Biasibetti et al. [181] demonstrated that long-term supplementation of EGCG with a dose of 10 mg/kg BW/day for four weeks, to the ICV-STZ induced rats, increased GPX activity by 105% as compared to control, and decreased NO and ROS levels generated in the hippocampus by 100% and 105% compared to control, respectively. Chronic exposure to the industrial chemical, such as acrylamide, could also result in neurotoxicity, whereby accumulation of acrylamide from dietary sources has been linked to mild cognition decline in elderly Chinese non-smoking men [182]. Again, treatment with ECGC at 20mg/kg BW/day for 14 days in rats attenuated the neurotoxic effect of acrylamide by significantly increasing the GSH level to 35 compared to 22 nmol/g tissue in untreated acrylamide-induced neurotoxicity rats in the cerebral cortex, which was comparable to the effects of vitamin E [183].

#### 4.4.4. Ginger Extract

Similar to curcumin, ginger is a family of Zingiberaceae that possesses potent antioxidant activity. It is a common spice used in cooking and traditional medicine. In Southeast Asia, four types of common tropical ginger have been identified and termed as galangal from different plant species, namely, *Alpinia galanga*, *Alpinia officinarum*, *Boesenbergia rotunda*, and *Kaempferia galanga*. Previous studies have experimented on the neuroprotective effects of the ethanolic extract from *A. galanga* (L) Willd on AD mice at the dose of 400 mg/kg BW/day for 14 days. The antioxidant enzymes of SOD were increased to 7.17 ± 0.51 vs. 1.82 ± 0.44 U/min/mg in negative control. In addition, GPX (33.41 ± 1.48, vs. 21.84 ± 0.66 U/min/mg) and CAT (2.16 ± 0.18 vs. 0.90 ± 0.10 U/mg) were also elevated, along with non-enzymatic antioxidant vitamin C (0.92 ± 0.03 vs. 0.55 ± 0.03 μg/mg after treatment with *A. galanga* (Table 3) [184]). Extracts of *A. galanga* could also inhibit MAO A and B activity at a low dose with a stronger inhibitory effect at a higher dose. MAO A activity was reduced to 18.10 ± 0.29 nmol/mg compared to 25.33 ± 0.45 nmol/mg in negative control, and similar results were observed with MAO B activity levels (17.60 ± 1.92 vs. 29.27 ± 0.66 nmol/mg). MAO is an enzyme that catalyzes the oxidative deamination of neurotransmitters including norepinephrine, serotonin, and dopamine, which have been suggested to increase H_2_O_2_ generation in the neurons. Collectively, the attenuation of free radicals by the enhanced endogenous antioxidant system and the inhibition of MAO enzymes by *A. galanga* improved cognition in AD mice [184].

Ginger contains gingerol, a phenolic compound involved in antioxidant and anti-inflammatory activity. Among all gingerols, 6-gingerols is the most abundant and associated with the pungent taste of ginger. The presence of β-hydroxy keto group in 6-gingerols makes the compound thermolabile and easily converts to 6-shogaols over dehydration and heating. Pre-treatment with 20 µM 6-shogaols to the H_2_O_2_ and 6-OHDA-induced cytotoxicity in PC 12 cells activated phase II enzyme genes expressions and corresponding protein activities. Induction of Nrf2 dependent genes, including *HO-1*, *TrxR1*, *NQO1*, *GCLC*, and the glutamate-cysteine ligase modifier subunit (*GCLM*), occurred as early as three hours post-treatment, where the relative mRNA expression of the respective genes were 14, 3, 2, 2, and 2. Subsequently, the protein expression of cognate proteins showed increased activity as well. The HO-1/actin, NQO1/actin, and TrxR1/actin ratio was 160, 175, and 145, respectively, in contrast to 100 in all of the control groups [185]. The 6-shogaols compound contains Michael acceptor in its chemical structure, which has been shown to interact with cysteine thiolate groups of Keap1, thus preventing the ubiquitination and degradation of Nrf2 [186]. Moreover, 6-shogaols treatment prompted the Nrf2 nuclear translocation. Treatment of 6-shogaols to the Nrf2 knockout PC 12 cells did not yield any results, indicating that the antioxidant protection of 6-shogaols is dependent on the Nrf2/ARE pathway [185].

#### 4.4.5. Vitamin E

The beneficial effects of vitamin E in alleviating oxidative stress have been documented in preclinical and clinical studies. Older individuals displayed an elevated risk of oxidative stress due to the decline in antioxidative status, which was associated with various chronic diseases [187]. TRF supplementation for 150 mg/day in older adults for six months markedly increased the SOD activity to 0.130 from 0.118 U/mL at baseline. There was also a significant increase in GPX activity in female subjects compared to baseline (2250 vs. 1750 nmol/mL/ min, respectively; *p* < 0.05) and 2.5-fold elevation of GSH/GSSG ratio after TRF supplementation (Table 3) [188]. It could enhance the GSH pool via several mechanisms. Firstly, vitamin E promotes GSH synthesis by increasing the bioavailability of the GSH precursor, γ-glutamylcysteine [189]. Additionally, vitamin E could stimulate GR enzyme activity in the brain resulting in a more efficient conversion of GSSG back to GSH [190]. Older rats that received 200 mg/kg BW/day of TRF for three months showed significant elevation of SOD, CAT, and GPX activity compared to aged rats that did not receive TRF (4 vs. 3 U/mg; 250 vs. 200 nmol/min/mg; 90 vs. 65 nmol/min/mg, respectively; *p* < 0.05). Moreover, due to its lipophilic nature, there was a higher concentration of total vitamin E in the brain of older rats (11.5 µg/mL) compared to age-matched controls (10 µg/mL), which reversed the cognitive deficits [113].

## 5. Oxidative Stress and Intracellular Signaling Pathway

### 5.1. Inflammatory Pathway

#### 5.1.1. Microglia and Astrocyte Activation

Oxidative stress is highly associated with inflammation, creating a vicious cycle between these two events in neurodegenerative diseases. Activated microglia and astrocytes are capable of secreting inflammatory cytokines, including interleukin (IL)-1, IL-6, IL-12, and IL-23, interferon-γ (IFN-γ), and TNFα, in addition to chemokines such as monocyte chemoattractant protein 1 (MCP1) and proteases [191]. As the Aβ is building up, microglia and astrocytes are activated, promoting the release of these pro-inflammatory responses. In addition to the production of pro-inflammatory substances, activated microglia and astrocytes could also produce a large amount of ROS and RNS due to stimulation of NOX and NOS, which further exacerbate neuronal damage. Chronic activation of these cells produces disproportionate amounts of neurotoxic inflammatory molecules that lead to inflammatory-mediated neuronal death [192].

#### 5.1.2. MAPK Pathway

In oxidative stress, ROS induced inflammatory responses mediated by the mitogen-activated protein kinase (MAPK) signaling pathway [193]. MAPKs are a group of protein-serine and threonine kinases that modulate cellular activity and functions in response to a wide variety of stimuli. MAPK consists of c-Jun *N*-terminal kinase (JNK), extracellular signal-regulated kinases (ERK), and the p38 MAPKs [194]. Activated MAPKs phosphorylate various proteins, including transcription factors, resulting in the promotion of inflammatory responses [195]. The MAPK signaling pathway is also vital in cellular growth, differentiation, survival, and death [196].

ROS have been shown to induce ERK pathway activation independent of ligand binding to its receptor tyrosine kinases (RTK) [197]. In the JNK and p38 pathway, thioredoxin (TRX) binding at N-terminal of apoptosis signal-regulating kinase 1 (ASK1) renders the protein inactivated. ROS could oxidize the TRX, causing it to dissociate from ASK1. Freed ASK1 becomes activated by forming oligomerization and autophosphorylation, subsequently, activating the JNK and p38 pathway [198]. Studies reported that p38 MAPK was upregulated in rodent models of AD, while in microglial cell culture, exposure to fibrillar Aβ promotes p38 MAPK phosphorylation, as well as activation of ERK pathway [199]. Active JNK is also reported in AD patients, animal models of AD, and involved in tau protein phosphorylation [200]. Treatment with SP600125, a specific JNK inhibitor, improved neuroinflammatory responses, and attenuated loss of synaptic function in rodent models of AD [201].

#### 5.1.3. NF-κB Pathway

Nuclear factor kappa-light-chain-enhancer of activated B cells (NF-κB), is a family of inducible transcription factors that target genes involved in inflammation development and progression. Moreover, it also mediates cell proliferation, differentiation, and survival. The NF-κB is located in the cytoplasm as a p50/p65 heterodimer, one of the NF-κB subunit, and is inhibited by an inhibitor of NF-κB (IκB) proteins. IκB kinase (IKK) phosphorylates IκB, dissociating it from p50/p65 heterodimer. In the nucleus, the released p50/p65 heterodimer binds to NF-κB response element in the DNA regulating expressions of target genes, including pro-inflammatory cytokines, adhesion molecules, and inflammatory enzymes [202]. IL-1β and TNFα, which are cytokines regulated by NF-κB, were increased in brains of AD patients and is mediated through microglial NF-κB stimulation by Aβ [203]. Moreover, NF-κB also plays a role in amyloid plaque formation, where increased NF-κB upregulates β-secretase enzyme 1 (BACE1), a protein that cleaves APP to generate Aβ [204]. Similar to AD, there is an increased in TNFα, IL-1β, IL-2, and IL-6 in brains and cerebrospinal fluid of PD patients [205]. ROS, such as H_2_O_2_, are also potent NF-κB activators, leading to pro-inflammatory conditions [206,207]. Taken together, NF-κB is an essential contributor to neuroinflammation, plaque progression, and neuronal death in AD and PD [208,209,210]

### 5.2. Cell Survival and Apoptotic Pathways

#### 5.2.1. PI3K/AKT Pathway

Phosphatidylinositol-3-kinases (PI3Ks) and Akt pathway is an intracellular signaling pathway essential in cell survival, growth, and proliferation following stimulation from the extracellular signal. When a ligand induces RTK autophosphorylation, PI3K is activated. PI3K initiates the pathway by phosphorylating phosphatidylinositol trisphosphate 2 (PIP2) to PIP3, a lipid second messenger on the plasma membrane [211]. The phosphorylated PIP3 recruit phosphoinositide-dependent kinase-1 (PDK1) that phosphorylates Akt, of which the downstream effects include cell proliferation and inhibition of apoptosis. Among the targets of Akt are forkhead boxO1 (FoXO1), mammalian target of rapamycin (mTOR), and glycogen synthase kinase-3β (GSK-3β) [212]. Akt inhibits GSK-3β by phosphorylating at serine-9 of GSK-3β [213]. Overactivity of GSK-3β promoted tau hyperphosphorylation and amyloid plaque formation [214]. Aβ oligomers can also inhibit the PI3K/Akt pathway, activating GSK-3β, causing neurotoxicity [215]. Meanwhile, in PD, there is a significant decrease of Akt expression with the loss of dopaminergic neuron compared to control [216]. It has been demonstrated that oxidative stress has impacts on the PI3K/Akt pathway. Cao et al. [217] reported that ROS repressed the PI3K/AKT pathway, through direct Akt dephosphorylation at Ser-473.

#### 5.2.2. Extrinsic Apoptotic Pathway

Oxidative stress is highly associated with increased apoptosis. There are two apoptotic pathways, namely the extrinsic and intrinsic pathway. Under physiological conditions, activation of the extrinsic pathway involves binding of Fas ligand to Fas ligand-receptor or binding of TNFα to TNF receptor 1(TNFR1) [218]. In both processes, the Fas-associated death domain (FADD) will be activated. It converts procaspase-8 into active caspase-8, which in turn converts procaspase-3 into caspase-3, the final executor of apoptosis [219]. In addition, the extrinsic apoptotic pathway can crosstalk with the intrinsic apoptotic pathway via caspase-8 [220].

#### 5.2.3. Intrinsic Apoptotic Pathway

The intrinsic apoptotic pathway or apoptosome mediated pathway begins intracellularly, in response to internal stimuli such as DNA damage and oxidative stress. In stressed conditions, activated p53, a tumor suppressor protein, promotes Bcl-2-associated X protein (Bax) expression, which leads to the formation of pores in the mitochondrial membrane [221]. These pores facilitate cytochrome c (Cyt c) release into the cytosol. Cyt c then binds to apoptotic protease-activating factor-1 (Apaf-1), and forms an apoptosome complex, in turn, the complex converts procaspase-9 into active caspase-9 [222]. Caspase-9 will further activate caspase-3, which leads to nuclear degradation and apoptosis. At the same time, p53 activates proteases for protein degradation and downregulates B-cell leukemia lymphoma 2 (Bcl-2), an inhibitor of Bax protein, overall aiding the apoptotic process [223].

### 5.3. Modulation of Inflammatory, Survival, and Apoptotic Signaling Pathways by Nutraceuticals

It is well established that an increased inflammation response and alteration to cell signaling pathways are among the primary cause that drives the progression of age-related neurodegenerative diseases. An increasing number of publications has shown that nutraceuticals are able to modulate these pathways. Hence, the investigations of nutraceuticals on neurodegenerative diseases have expanded beyond the antioxidant properties and target the intracellular signaling pathways.

#### 5.3.1. Curcumin

The use of LPS in animal and cell study has resulted in an increased ROS, microglia activation, cytokine production, and eventually neuronal death. In a study by Khan et al. [224], curcumin at a dose of 300 mg/kg BW/day was given to LPS-induced neurotoxicity rats for 14 days to assess its anti-inflammatory property. Treatment with curcumin significantly decreased the cytokine production TNF-α (relative density: 1.4 vs. 2.0 AU) and IL-1β (1.8 vs. 2.5 AU) by reducing the p-NF-κB (1.2 vs. 2.8 AU) and p-JNK expression (density of values: 48,000 vs. 32,000 AU) in the rat’s hippocampus compared to untreated rats (all *p* < 0.05) (Table 4) [224]. Furthermore, the inhibitory effect of curcumin on p-JNK was similar to SP600125 (density value: 0.6 vs. 1.2 AU, *p* > 0.05), when observed in vitro [224]. The LPS toxin stimulates the generation of ROS, which in turn stimulates the JNK pathway and initiates downstream neuroinflammatory cascades. At the same time, LPS downregulates the survival pathway p-Akt/p-GSK-3β via activated microglia. Curcumin inhibited the overexpression of glial fibrillary acidic protein (GFAP) (relative density: 4.4 vs. 5.4 AU, *p* < 0.05), and ionized calcium binding adaptor molecule 1 (Iba-1) (relative density: 1.4 vs. 2.4 AU, *p* < 0.05) in the hippocampus of LPS-induced rats and restored the survival pathway [224]. The anti-apoptotic ability of curcumin was also demonstrated in this study where apoptotic markers including Bax (density of values: 1.6 vs. 4.0 AU), caspase-3 (4.0 vs. 6.0 AU), Cyt c (1.6 vs. 2.0 AU), and Poly (ADP-ribose) polymerase-1 (PARP-1)) were reduced (6.0 vs. 7.0 AU), while an anti-apoptotic protein, Bcl-2, was increased (0.6 vs. 0.4 AU) in the hippocampus (all *p* < 0.05) [224].

Ar-turmerone, another primary bioactive compound of *Curcuma longa*, displayed protective effects against neuroinflammation. Compared to control, Aβ-induced activation of microglia resulted in increased generation of MCP1 (300 vs. 10 pg/mL), TNF-α (150 vs. 10 pg/mL), IL-1β (300 vs. 20 pg/mL), and IL-6 (300 vs. 30 pg/mL), which were attenuated with ar-turmerone treatment at 20 μM (MCP-1: 100 pg/mL; TNF-α: 50 pg/mL; IL-1β 50 pg/mL; and IL-6: 50 pg/mL; all *p* < 0.05) [225]. Correspondingly, ar-turmerone suppressed signaling pathways that are involved in inflammation, including the NF-κB and MAPK pathways. Ar-turmerone at similar dose inhibited both Aβ-induced IκB phosphorylation and degradation as well as nuclear translocation of the NF-κB p65 subunit. Additionally, ar-turmerone prevented p-JNK and p38 kinase activation, overall reducing neuroinflammation mediated by activated microglia cells (relative densities of p-IκB, p65, p-JNK, and p38 were shown but not quantified) [225].

Peroxisome proliferator-activated receptors gamma (PPARγ) can inhibit inflammatory responses in the AD brain via downregulation of the NF-κB and ERK pathways. In this regard, intraperitoneal curcumin at 150 mg/kg BW/day for four consecutive weeks has been shown to act as a PPARγ agonist in APP/PS1 mice, a transgenic mice model of AD that expressed elevated Aβ-protein level deposition [226]. There was a two-fold increase in PPARγ transcriptional activity as well as PPARγ protein expression after treatment with curcumin both in vitro (PPARγ activity: 4.0 vs. 2.0 AU; protein expression: 0.9 vs. 0.3 AU) and in vivo models of AD (PPARγ activity: 4.5 vs. 2.0 AU; protein expression: 1.0 vs. 0.3 AU) [226]. Together with this, the markers of neuroinflammation, such as IL-1β, TNF-α, and cyclooxygenase-2 (COX-2), were significantly attenuated via curcumin activation on PPARγ [226].

Neuroinflammation is also initiated as a response from the activated innate immune system. This includes the Toll-like receptor 4 (TLR4) and receptor for advanced glycation end products (RAGE) that are highly expressed in activated microglia [227]. The binding of pathogen-associated molecular patterns (PAMPs) and damage-associated molecular patterns (DAMPs) to TLR4 and RAGE activate the NF-κB signaling pathway and induce inflammation [228]. In mice with ethanol-induced oxidative stress, curcumin treatment at 50 mg/kg BW daily for six weeks decreased the elevated expressions of TLR4 (relative density: 1.0 vs. 2.2 AU), RAGE (1.0 vs. 2.2 AU), GFAP (1.4 vs. 2.3 AU), and Iba-1 (1.0 vs. 2.8 AU) compared to untreated mice (all *p* < 0.05) [229]. In addition, curcumin abrogates the phosphorylation of JNK (1.6 vs. 2.1 AU) and its downstream targets, including p-NF-κB, IL-1β, Cox-2, and TNF-α, providing potent neuroinflammatory protection [229].

In a study evaluating the early-onset PD, curcumin was shown to increase cell survival mechanisms in neuroblastoma cells overexpressing A53T, α-Syn proteins with point mutations at the 53rd amino acids sequence. Macroautophagy is one of the cell survival mechanism where degradation of intracytoplasmic aggregate-prone proteins occurs. Macroautophagy can be controlled with the direct mTOR pathway or indirectly via the inhibition of autophagy-related protein 1 (Atg1) by the mTOR/p70S6K pathway. Phosphorylated 70S6K, a ribosomal protein S6 kinase, is a substrate of mTOR that inhibits autophagy. Dysfunction in the autophagy pathway led to the accumulation of α-Syn in PD. Compared to untreated baseline cells, curcumin treatment at 6 µM in vitro inhibited α-Syn A53T-induced mTOR (mTOR/actin: 0.7 vs. 2.0) and p70S6K activity (p70S6K/actin: 0.6 vs. 1.7), hence, this increased the clearance of A53T α-syn (α-Syn/actin: 40 vs. 120) by restoring the autophagy pathway marker LC3 (LC3/actin: 0.7 vs. 0.3) (all *p* < 0.05) [230].

Demethoxycurcumin is a derivative of curcumin, found in *Curcuma zedoaria*, *Etlingera elatior*, and *Curcuma longa*, and holds a wide variety of medicinal effects in neurological disorders. Treatment with demethoxycurcumin ameliorated the adverse effects of rotenone, a neurotoxic pesticide, on SH-SY5Y cells by decreasing ROS (fluorescent dichlorofluorescein intensity: 3.5 vs. 5.0%), preserving mitochondrial membrane potential (fluorescent dye Rhodamine-123 intensity: 80 vs. 60%), and inhibiting cell death (cell viability: 90 vs. 60%) (all *p* < 0.05) [92]. Cells treated with 50 nM demethoxycurcumin displayed an increase in the anti-apoptotic protein expression, including Bcl-2 (relative intensity: 80- vs. 50-fold of control) and Bcl-xL (70- vs. 60-fold), while decreasing pro-apoptotic protein expressions, including Bcl-2-associated death promoter (BAD) (130- vs. 160-fold), caspase-3 (140- vs. 210-fold), caspase-6 (130- vs. 170-fold), caspase-8 (130- vs. 170-fold), and caspase-9 (140- vs. 190-fold), in the mitochondria (all *p* < 0.05) [92]. 

#### 5.3.2. Green Tea Polyphenols

EGCG exerts neuroprotective mechanism via activation of protein kinase C (PKC). PKC is involved in the signaling pathway that promotes cell differentiation, growth, and survival [231]. In 6-OHDA-induced oxidative stress of neuroblastoma SH-SY5Y cells, EGCG pre-treatment at 1 μM increased cell survival (70 vs. 35%) by upregulating the phosphorylated PKC (relative density: 11- vs. 5-fold) (both *p* < 0.05) compared to untreated 6-OHDA-exposed cells [232]. This showed that the increased in cell survival is dependent on PKC, as the addition of “GF109203X” (bisindolylmaleimide I), a PKC inhibitor, reversed the neuroprotection effects of ECGC in 6-OHDA toxicity. The activated PKC further stimulated the ERK1/2 signaling pathway (relative density: 0.9- vs. 0.6-fold), which improved the overall cell survival capacity (Table 4) [232].

In an in vivo study, EGCG treatment at 2.5 mg/kg BW daily for two weeks induced hippocampal neurogenesis (net BrdU+/NeuN+ cells: 900 vs. 500) in young male Balb/C mice by increasing the phosphorylated Akt (relative density: 450% of control) (both *p* < 0.001) [233]. Inhibition of PI3K, an upstream of Akt by its specific inhibitor LY294002, further supported the involvement of PI3K/Akt pathway to induce neuronal differentiation and survival (percentage of MAP2 from Dapi cells, EGCG vs. LY+EGCG: 25 vs. 14%) [233]. The antiapoptotic effect of green tea polyphenols was further investigated in glutamate excitotoxicity in primary cultured cortical neurons. Pretreatment with 10 μM green tea polyphenols to glutamate-exposed primary cortical neurons decreased the expression of proapoptotic proteins Bax (relative density, Bax/actin: 100 vs. 130%) and caspase-3 (caspase-3/actin: 120 vs. 170%) (both *p* < 0.05), while the antiapoptotic protein Bcl-2 increased (Bcl-2/actin: 100 vs. 70%, *p* < 0.01) [234].

#### 5.3.3. Berberine

Berberine is an isoquinoline alkaloid compound found in several plants species, including *Berberis vulgaris*, *Berberis aristate*, *Mahonia aquifolium*, and *Xanthorhiza simplicissima* [235]. It is traditionally used in Chinese herbal medicine as an anti-diabetic agent, and later, its benefit expanded to treat a wide variety of diseases, including cardiovascular, renal, and central nervous system disorders [236]. The protective effects of berberine in modulating the Aβ-induced neuroinflammatory response have been demonstrated. Pre-treatment of Aβ-exposed microglial cells with 5 μM berberine inhibited IκB phosphorylation (relative density, p-IκBα/β-actin: 0.4- vs. 1.2-fold of control) at the key serine Ser32/36 sites [237]. This process led to an inhibition of NF-κB p65 activation, showed as reduced p65 phosphorylation. As a result, accumulation of phosphorylated p65 was markedly reduced in the berberine-treated compared to untreated Aβ-exposed BV2 cells, a cell line derived from the murine microglial cell that acts as a substitute for primary microglial cells (Table 4) [237]. Berberine was also involved in MAPK and Akt inflammatory pathway in which Aβ-induced phosphorylation of Akt, (relative density, p-Akt/Akt: 1.0- vs. 1.3-fold of control), p38 (p-p38/p38: 0.5- vs. 2.5-fold), and ERK (p-ERK/ERK: 0.3- vs. 1.7-fold) was prevented in a dose-dependent manner, with maximal effects exerted at 5 μM (all *p* < 0.05) [237].

#### 5.3.4. Magnolol

The anti-inflammatory effects of magnolol in AD are partly due to its activation on PPAR-γ. PPAR-γ activation led to the inhibition of the DNA-binding activity of NF-κB hence attenuated inflammation response. A concentration of 2.5 µM of magnolol significantly inhibited the transcriptional activity of NF-κB in the HEK293 T cells, a cell line derived from the human embryonic kidney that were transfected with NF-κB reporter plasmids [238]. The relative luciferase activity of NF-κB in magnolol treated cells (3.5-fold) was significantly lower compared to control (5.5-fold). In addition, magnolol markedly reduced the mRNA expressions of the downstream targeted genes by NF-κB such as iNOS (0.75), TNF-α (0.75), and IL-1β (0.55) in BV2 cells that were pre-treated with oligomeric Aβ42 (*p* < 0.05). The anti-inflammatory effects of magnolol in this study were reversed with the addition of GW9662, a potent antagonist of PPAR-γ [238].

Apart from that, magnolol has been shown to exhibit neuroprotective effects against acrolein-induced apoptosis in neuroblastoma SH-SY5Y cells via the PI3K/MEK/ERK and PI3K/Akt/FoxO1 signaling pathways, in which the effects appeared to be upstream to PI3K [96]. Treatment of magnolol at 16 µM on acrolein-induced apoptosis SH-SY5Y neuroblastoma cells significantly attenuated apoptosis at 10%, while the addition of a specific PI3K inhibitor LY294002 increased the apoptosis to 60%, which was similar to the acrolein-exposed cells [96]. Magnolol treatment inhibited phosphorylation of MEK and ERK, while it promoted phosphorylation of Akt and FoXO1, which confirmed the involvement of previously mentioned pathways.

#### 5.3.5. Vitamin E

Previous studies have shown that vitamin E could alleviate neurodegenerative diseases by regulating gene expression in the hippocampus. Daily long term supplementation with 200 mg/kg BW of TRF in APPswe/PS1dE9 AD mouse model for six months caused significant downregulation of 3615 genes compared to AD control mice that included decreased expressions of the *Hdac2* and *Iqgap2* genes, which were involved in the inflammatory process via TNF-α/NF-κB signaling pathway [239]. Apart from that, TRF decreased expression of pro-apoptotic genes, including *Casp7*, *Casp8*, *Casp12*, and *Bid* that might result in apoptosis inhibition in the neurons [239]. Additionally, supplementation with TRF might limit calcium entry into the neurons by regulating the voltage-dependent calcium channel genes, including *Cacna1c* and *Cacna1d* (Table 4). This process was crucial, since calcium overload could stimulate the opening of mitochondrial permeability transition pore resulting in cytochrome c release and eventually apoptosis [240]. 

## 6. Conclusions

**Figure 1:**
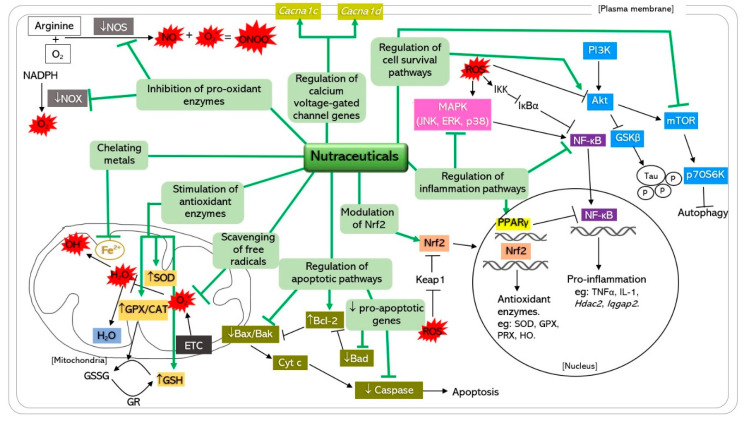
The proposed mechanism of action of nutraceutical in alleviating neurodegenerative disease. Abbreviations: Akt: protein kinase B; Bad: BCL 2 associated agonist of cell death; Bax: Bcl-2-associated X protein; Bak: BCL2 Antagonist/Killer; Bcl-2: B-cell lymphoma 2; *Cacna1c*: calcium voltage-gated channel subunit alpha1 C genes; *Cacna1d*: calcium voltage-gated channel subunit alpha1 D genes; caspase: cysteine-aspartic proteases; CAT: catalase; Cyt c: cytochrome C; ETC: electron transport chain; ERK: extracellular signal-regulated kinase; Fe^2+^: ferrous ion; GPX: glutathione peroxidase; GSH: glutathione; GSK-3β: glycogen synthase kinase-3β; GSSG: oxidized glutathione; GR: glutathione reductase; *Hdac2*: genes that encode histone deacetylase 2; HO: heme oxygenase; H_2_O_2_: hydrogen peroxide; IKK: IκB kinase; IκBα: nuclear factor kappa-light-chain-enhancer of activated B cells inhibitor, alpha; IL-1: interleukin-1; *Iqgap2*: genes that encode ras GTPase-activating-like protein; JNK: c-Jun N-terminal kinases; Keap 1: Kelch-like ECH-associated protein 1; MAPK: mitogen-activated protein kinase; mTOR: mammalian target of rapamycin; NF-κB: nuclear factor kappa-light-chain-enhancer of activated B cells; NADPH: reduced form of nicotinamide adenine dinucleotide phosphate; Nrf2: nuclear factor erythroid 2–related factor 2; NOS: nitric oxide synthases; NO: nitric oxide; NOX: NADPH oxidase; O_2_^•−^: superoxide anion; OH^•^: hydroxyl radical; O_2_: oxygen; ONOO^−^: peroxynitrite; PI3K: phosphoinositide 3-kinase; PPARγ: peroxisome proliferator-activated receptor gamma; PRX: peroxiredoxin; p70S6K: a ribosomal protein S6 kinase; ROS: reactive oxygen species; SOD: superoxide dismutase; TNFα: tumor necrosis factor alpha; ↑: increased; ↓: decreased.

The search for novel therapeutic agents in combating neurodegenerative diseases is still actively studied, with a particular interest in nutraceuticals. The proposed mechanisms of action of nutraceuticals on the neurodegenerative diseases are summarized in (Figure 1). Nutraceuticals have been shown to act as a potent antioxidative agent both in in vivo and in vitro studies. Nutraceuticals possess direct antioxidation properties by scavenging the reactive oxidative and nitrosative molecules. Additionally, it could activate the gene and protein expressions of various antioxidation enzymes as well as deactivate pro-oxidant enzymes. It could also inhibit programmed cell death by blocking both the extrinsic and intrinsic apoptotic pathway, besides providing an anti-inflammatory response. Regulation of apoptosis, inflammation, and oxidative stress could be supported by the nutraceutical’s ability to regulate various intracellular signaling pathways. Furthermore, metal chelation properties of nutraceuticals provide further mechanism in alleviating oxidative stress in neurodegenerative diseases. In conclusion, antioxidant nutraceuticals offer promising outcome against neurodegenerative diseases.

## Figures and Tables

**Table 1:** Direct antioxidation properties of nutraceuticals.

Nutraceutical and Its Active Compounds	Dosage	Experimental Models	Direct Antioxidant Effects	References
Resveratrol	10 mg/kg BW/day for two weeks	Angiotensin II-induced early AD rats	↓ superoxide levels in the nucleus tractus solitarius and hippocampus.	[69]
	10 and 20 mg/kg BW for 21 days	ICV-STZ infused rats	↓ MDA levels in the brain.	[72]
Piceatannol	10 and 20 µM	Aβ-induced cytotoxicity PC 12 cells	↓ the intracellular ROS generation.	[71]
Grape seed extract. Active compound: Proanthocyanidins	100 mg/kg BW/day for 30 days	Aged rats	↓ 8-OHdG and DNA protein cross-links levels in the rat brains and spinal cords.	[76]
			↓ ROS and PC levels in the rat brains and spinal cords.	[77]
EGb 761 extract. Active compounds: 24% flavonols (kaempferol, quercetin and isorhamnetin), 6% terpene lactones (ginkgolide-A, -B, -C -M, and -J and bilobalide B)	Pre-treatment of EGb 761 (100 µg/mL) for 48 h	Aβ-secreting mutant cells	↓ intracellular ROS levels.	[29]
Green tea polyphenols	2 g/day of green tea pills. Each pill contained 50 mg of total polyphenols (EGCG, EC, and ECG).	Severe AD patients	Levels of MDA, 8-OHdG and PC ↓ significantly as compared to baseline values.	[83]
			↑ FRAP assay.	
Curcumin	2000 parts per million (ppm) of curcumin mixed in the diet	Aβ-induced cognitive deficits and neuropathology rats	↓ IsoPs levels in rat brains.	[84]
	500 ppm of curcumin mixed in the diet	APP transgenic mouse	↓ LPO in the spinal cord.	[85]
CUR-CA-THIONE	500 mg/kg BW/day for fifteen days	Aluminum chloride-induced AD rats	Inhibits LPO in the rat brains.	[91]
Demethoxycurcumin	50 nM	Rotenone induced neurotoxicity in neuroblastoma cells	↓ the intracellular ROS generation.	[92]
Xanthorrhizol	0.5, 1, 5, and 10 µM	H_2_O_2_-induced lipid peroxidation in rat brain homogenate	↓ LPO in the rat brains.	[93]
	2 µM	Glutamate-induced cytotoxicity in hippocampal neurons	↓ intracellular ROS generation in the hippocampal neurons.	
Magnolol	30 mg/kg BW, given once after MPTP injection	MPTP-induced Parkinson in mice	↓ lipid peroxidation in the striatum.	[94]
	16 and 32 µM	Acrolein-induced oxidative damage in neuroblastoma cells	↓ PC, HNE, and superoxide levels.	[96]
Pycnogenol^®^. Active compounds: Procyanidins, phenolic acids, and polyphenols (catechin, epicatechin, and taxifolin)	50 and 100 μg/mL	Acrolein-induced oxidative damage in neuroblastoma cells	↓ ROS, superoxide, PC, and 4-HNE levels.	[99]
	Pre-treatment of Pycnogenol^®^, 10 mg/kg BW for three weeks	ICV-STZ induces AD in rats	↓ PC and LPO in the hippocampus and cerebral cortex.	[100]
	150 mg/day for six weeks	Healthy subjects	↑ antioxidant capacity of plasma compared to baseline.	[101]
	50 mg/day for eight weeks	Human subjects (smokers)	↓ plasma reactive oxygen metabolites and ↑ antioxidant potential compared to smokers taking placebo.	[102]
Guarana seed extract. Active compound: Polyphenols (catechins and epicatechins)	100 and 1000 μg/mL	Acrolein-induced cytotoxicity on neuronal cells	↓ intracellular ROS production.	[108]
	10 and 50 mg/mL of GHE	*C. elegans* models of AD		[107]
Tocotrienol rich fraction (TRF). Active compounds: α-tocopherol (23.40%) and α-, β-, γ-, and δ-tocotrienol (27.30, 3.34, 35.51, and 10.45%, respectively)	150 mg/day for six months	Older adults aged 50–55 years old	↓ plasma MDA and DNA damage.	[115]
	200 mg/kg BW for eight months	APPswe/PS1dE9 transgenic mouse model of AD	↓ DNA strand breaks in the blood.	[117]

Aβ: amyloid beta; AD: Alzheimer disease; APP: amyloid precursor protein; APPswe/PS1dE9: a transgenic AD mouse model that displays elevated Aβ plaque due to Swedish mutations in APP and deleted presenilin-1 in exon 9; BW: body weight; *C. elegans*: *Caenorhabditis elegans*; CUR-CA-THIONE: curcumin concoction using glutathione and casein as vectors; EC: epicatechin; ECG: epicatechin-3-gallate; EGC: epigallocatechin; EGCG: epigallocatechin-3-gallate; EGb 761: standardized herbal extract from the *Ginkgo biloba* plant; FRAP: ferric reducing antioxidant power; GHE: guarana hydroalcoholic extract; 4-HNE: 4-hydroxy-2-nonenal; H_2_O_2_: hydrogen peroxide; ICV-STZ: intracerebroventricular-streptozotocin; IsoPs: isoprostanes; LPO: lipid peroxides; MDA: malondialdehyde; MPTP: 1-methyl-4-phenyl-1,2,3,6-tetrahydropyridine; MPP^+^: 1-methyl-4-phenylpyridinium; PC: protein carbonyls; PC 12 cells: a cell line derived from pheochromocytoma in rats; Pycnogenol^®^: standardized extract exclusively from the bark of French maritime pine; ROS: reactive oxygen species; TRF: tocotrienol rich fraction; 8-OHdG: 8-hydroxy-2′-deoxyguanosine; ↑: increased; ↓: decreased.

**Table 2:** Metal chelation activities of nutraceuticals.

Nutraceutical and Its Active Compounds	Dosage	Experimental Models	Metal Chelators Effect	References
EGCG	20 µM	Fe^3+^ induced fibrillation of α-Syn and Fe^3+^ induced cytotoxicity in α-Syn-PC 12 cells	Molecular: Slowed the formation of α-Syn fibrillation and β-sheet conformers, inhibited the conformational transition of α-Syn in the nucleation period, remodel amyloid fibrils into soluble amorphous aggregates.	[137]
			Cellular: ↓ ROS generation and ↑ cell viability.	
	20 µM	Cu^2+^ induced fibrillation of α-Syn and Cu^2+^ induced cytotoxicity in α-Syn-PC 12 cells	Molecular: inhibited the formation of Cu^2+^-induced amyloid fibrillation of α-Syn, inhibited β-sheet conformation, restructure amyloid fibrils into soluble amorphous aggregates, forming EGCG/Cu^2+^ complex and binds to Tyr in α-Syn.	[139]
			Cellular: ↓ expression and aggregation of α-Syn, cell apoptotic percentage, ↓ ROS generation.	
	5 and 10µM	SH-SY5Y cells	↑ the TfR and TfR mRNA expressions as well as ↓ APP expression.	[141]
Curcumin	In vitro: 12.5, 25 and 50 µM	Liver cell line BNL CL.2 and mice fed with 2% of dietary curcumin	↓ H and L ferritin protein expression.	[142]
	In vivo: 2.0% dietary curcumin for twelve weeks			
	200 mg/kg BW/twice a day for twenty-four days	6-OHDA-induced PD in Wistar rats	↓ the number of iron positive cell count in substantia nigra.	[143]
	25 µM	Wistar brain homogenate	↓ Fe^2+^ and Quinolic acid-induced lipid peroxidation.	[144]
	2:1 and 1:1 curcumin-Cu^2+^ complexes; 2:1 and 1:1 curcumin-Zn^2+^ complexes	H_2_O_2_-induced injury in PC 12 cells	↑ antioxidant enzymes, CAT, SOD and GPX levels.	[145]
			↓ ROS and MDA levels and ↑ cell viability.	

APP: amyloid precursor protein; α-Syn: α-Synuclein; BNL CL.2:a cell line derived from murine embryonic liver cells; BW: body weight; CAT: catalase; Cu^2+^: cupric ion; Fe^3+^: ferric ion; EGCG: epigallocatechin gallate; GPX: glutathione peroxidase; H ferritin: heavy ferritin; H_2_O_2_: hydrogen peroxide; L ferritin: light ferritin; MDA: malondialdehyde; mRNA: messenger ribonucleic acid; PC 12 cells: a cell line derived from pheochromocytoma of the rat adrenal medulla; PD: Parkinson disease; ROS: reactive oxygen species; SH-SY5Y: human-derived neuroblastoma cell line; SOD: superoxide dismutase; TfR: transferrin receptor; Tyr: tyrosine; 6-OHDA: 6-hydroxydopamine; ↑: increased; ↓: decreased.

**Table 3:** Indirect antioxidation properties of nutraceuticals.

Nutraceutical and Its Active Compounds	Dosage	Experimental Models	Indirect Antioxidant Effects	References
Resveratrol	12.5 mg/kg BW/day for seven days	Healthy Wistar rats	↑ CAT, SOD, and peroxidase in rat brains.	[168]
	50 µM	Cultured lymphoblastoid cell lines of AD patients	↑ *CAT*, *SOD2*, and *NFE2L2* gene expressions.	[169]
	15 µM	H_2_O_2_ induced cytotoxicity on PC 12 cells	↑ *HO-1* and *GCLC* gene expression and stimulated Nrf2 nuclear translocation.	[171]
Curcumin	20 µmol/L	MPP^+^ induced cytotoxicity on PC 12 cells	↓ iNOS overexpression, and attenuated MPP+ induced apoptosis.	[172]
	5, 10, and 15 µM	Nrf2^+/+^ primary astrocytes with H_2_O_2_ insult	↑ Nrf2 target genes (HO-1, NQO1, GCLC) in Nrf2^+/+^ primary astrocytes.	[174]
	100 mg/kg BW/twice a day for 50 days	Rotenone-induced PD rats	↑ GSH, HO-1 and NQO1 protein through the Akt/Nrf2 pathway.	[175]
	15, 20, and 30 µM	Iodoacetate induced cytotoxicity on primary rat cerebellar granule neurons	↑ HO-1 expression.	[176]
	5 µM	C6 rat glioma cells	↑ genes involved in antioxidant response including *HO-1*, NQO1, *CAT*, *TrxR*, and *GCLC.*	[177]
EGCG	2 mg/kg BW/day for 10 days	MPTP-induced parkinsonism in male C57/BL mice	Prevented the ↑ of SOD and CAT in the striatum induced by MPTP.	[178]
	10 and 50 mg/kg BW/day for 14 days	MPTP-induced parkinsonism mice	↓ iNOS expression in substantia nigra and striatum.	[179]
	1, 5, and 10 µM	Lipopolysaccharide-induced microglial activation	↓ iNOS in activated microglia.	[180]
	10 mg/kg BW/day for four weeks	ICV-STZ infusion in rats	↑ GPX and maintained GSH as well as, ↓ ROS and NO levels in the hippocampus.	[181]
	20 mg/kg BW/day for 14 days	Acrylamide-induced neurotoxicity in rats and cytotoxicity in PC 12 cells	↑ GSH level in the cerebral cortex.	[183]
Ginger extract	200 and 400 mg/kg BW/day for 14 days	Aβ induced AD mice	↑ SOD, GPX, CAT, and non-enzymatic antioxidant vitamin C in the brain. Inhibited the MAO A and B activity.	[184]
6-shogaols	20 µM	H_2_O_2_- and 6-OHDA-induced cytotoxicity in PC 12 cells	↑ phase two enzymes genes expression and the respective enzyme’s activity; HO-1, NQO1, TrxR1, GCLC, and GCLM via activation of the NrF2/ARE pathway.	[185]
			↑ translocation of Nrf2 from the cytosol to nucleus	
Tocotrienol rich fraction (TRF). Active compounds: α-tocopherol (23.40%) and α, β, γ, and δ-tocotrienol (27.30, 3.34, 35.51, and 10.45%, respectively)	150 mg/day for six months	Healthy older adults aged 50 to 55 years	↑ SOD, GPX, and GSH/GSSG ratio in the blood compared to baseline	[188]
	200 mg/kg BW/day for three months	Aged Wistar rats (21 months old)	↑ SOD, CAT, and GPX in the blood. Higher concentrations of total vitamin E in the brain.	[113]

Akt: protein kinase B; Aβ: amyloid beta; AD: Alzheimer disease; ARE: antioxidant response element; BW: body weight; CAT: catalase; C6 rat glioma cell: a glioma cell line derived from a rat glial tumor induced by N-nitrosomethylurea; C57/BL mice: an inbred strains of mice; EGCG: Epigallocatechin gallate; GCLC: glutamyl-cysteine ligase; GCLM: glutamate-cysteine ligase modifier subunit; GPX: glutathione peroxidase; GSH: reduced glutathione; GSSG: oxidized glutathione; GSTZ1: glutathione S-transferase zeta 1; HO-1: heme oxygenase-1; H_2_O_2_: hydrogen peroxide; ICV-STZ: intracerebroventricular-streptozotocin; iNOS: inducible nitric oxide synthase; MAO A: monoamine oxidase A; MAO B: monoamine oxidase B; MPTP: 1-methyl-4-phenyl-1,2,3,6-tetrahydropyridine; MPP^+^: 1-methyl-4-phenylpyridinium; *NFE2L2*: nuclear factor, erythroid 2 like 2 gene; NO: nitric oxide; Nrf2: nuclear factor erythroid 2-related factor 2; Nrf2^+/+^: homozygous wild-type Nrf2^+/+^; NQO1: quinine oxidoreductase 1; PC 12 cells: a cell line derived from pheochromocytoma of the rat adrenal medulla; PD: Parkinson disease ROS: reactive oxygen species; SOD: superoxide dismutase; SOD1: copper-zinc-dependent superoxide dismutase; SOD2: manganese-dependent superoxide dismutase; TRF: tocotrienol rich fraction; TrxR: thioredoxin reductase; 6-OHDA: 6-hydroxydopamine; ↑: increased; ↓: decreased.

**Table 4:** Regulation of apoptosis, inflammation, and survival signaling pathways by nutraceuticals.

Nutraceutical and Its Active Compounds	Dosage	Experimental Models	Signaling Pathways Affected	References
Curcumin	300 mg/kg BW/day for 14 days	Lipopolysaccharide-induced neurotoxicity in rats and microglial cells	↓ p-JNK, p-NF-κB, TNF-α, IL-1β, GFAP, and Iba-1 in the hippocampus.	[224]
			↓ apoptotic markers (Bax, caspase-3, Cyt c, and PARP-1), while anti-apoptotic protein Bcl-2 was ↑.	
			Inhibited p-JNK activation similar to the JNK inhibitor, SP600125, in vitro.	
Ar-turmerone	20 μM	Aβ-stimulated microglial cells	↓ MCP1, TNF-α, IL-1β, and IL-6 by inhibiting MAPK (p-JNK and p38) and NF-κB signaling pathways.	[225]
Curcumin	150 mg/kg BW/day for four consecutive weeks	Transgenic APP/PS1 mice and mixed neuronal/glial cultures	↓ microglial activation as well as inflammatory mediators, including TNF-α, IL-1β, COX-2, and NO.	[226]
			Inhibited IκBα degradation and NF-κB p65 translocation.	
			↑ in PPARγ transcriptional activity and PPARγ protein expression.	
	50 mg/kg BW daily for six weeks	Ethanol-induced oxidative stress in mice brains	↓ TLR4, RAGE, GFAP, and Iba-1 expressions.	[229]
			↓ p-JNK, p-NF-κB, COX-2, IL-1β, and TNF-α.	
	6 µM	A53T α-syn cell model of Parkinsonism	Recovered autophagy via inhibition of the mTOR/p70S6K signaling pathway.	[230]
			↑ clearance of A53T α-syn accumulation.	
Demethoxycurcumin	50 nM	Rotenone induced neurotoxicity in SH-SY5Y neuroblastoma cells	↑ in the anti-apoptotic protein expression (Bcl-2 and Bcl-xL).	[92]
			↓ pro-apoptotic protein expressions (Bad, caspase-3, caspase-6, caspase-8, caspase-9).	
Epigallocatechin gallate	1 μM	6-OHDA-induced cytotoxicity in neuroblastoma cells	↑ PKC and ERK1/2 activities.	[232]
			↓ mRNAs expression of Bax, Bad, and Mdm2 while ↑ in Bcl-2, Bcl-w, and Bcl-xL.	
	2.5 mg/kg BW daily for two weeks	Balb/C mice	↑ neurogenesis in the hippocampus via the PI3K/Akt pathway.	[233]
Green tea polyphenols	10 μM	Glutamate excitotoxicity in primary cultured cortical neurons	↓ the expression of proapoptotic proteins Bax and caspase-3.	[234]
			↑ the antiapoptotic protein Bcl-2.	
Berberine	5 μM	Aβ-stimulated microglial cells	↓ MCP1 and IL-6 and their mRNA expressions.	[237]
			Inhibited NF-κB nuclear translocation by suppressing phosphorylation of IκB.	
			Inhibited phosphorylation of Akt, ERK1/2, and p38.	
Magnolol	2.5 µM	BV2 cells exposed to oligomeric Aβ42	↓ NF-κB target gene expressions including iNOS, TNF-α, and IL-1β.	[238]
	16 µM	Acrolein-induced apoptosis in neuroblastoma SH-SY5Y cells	↓ apoptotic cell via the PI3K/MEK/ERK and PI3K/Akt/FoxO1 signaling pathways.	[96]
Tocotrienol rich fraction (TRF). Active compounds: α-tocopherol (23.40%) and α, β, γ, and δ-tocotrienol (27.30, 3.34, 35.51, and 10.45%, respectively)	200 mg/kg BW/day for six months	APPswe/PS1dE9 AD mouse model	↓ *Hdac2* and *Iqgap2* genes involved in the NF-κB signaling pathway.	[239]
			↓ expression of pro-apoptotic genes (*Casp7*, *Casp8*, *Casp12*, and *Bid*).	
			Regulated the voltage-dependent calcium channel genes (*Cacna1c* and *Cacna1d*)	

α-Syn: α-Synuclein; Aβ: amyloid beta; Aβ42: amyloid beta 42; AD: Alzheimer disease; *Apaf1*: apoptotic peptidase activating factor 1 gene; APP/PS1: a transgenic mice model of AD that expresses elevated Aβ-protein level deposition; APPswe/PS1dE9: a transgenic AD mouse model that displays elevated Aβ plaque due to Swedish mutations in APP and deleted presenilin-1 in exon 9; Akt: protein kinase B; A53T: α-Syn proteins that have point mutations at the 53rd amino acids; Bad: Bcl-2 associated agonist of cell death; Bax: Bcl-2-associated X protein; Bcl-2: B-cell lymphoma 2; Bcl-xL: B-cell lymphoma-extra-large; Bcl-w: alias of Bcl-2-like protein 2; *Bid*: BH3 interacting-domain death agonist gene; BW: body weight; BV2: a cell line derived from the murine microglial cell that acts as a substitute for primary microglial cells; *Cacna1c*: calcium voltage-gated channel subunit alpha1 C gene; *Cacna1d*: calcium voltage-gated channel subunit alpha1 D gene; *Casp7*: Caspase-7 gene; *Casp8*: Caspase-8 gene; *Casp12*: Caspase-12 gene; COX-2: cyclooxygenase-2; Cyt c: cytochrome c; p-: phosphorylated; ERK: extracellular signal-regulated kinase; FoXO1: forkhead boxO1; GFAP: glial fibrillary acidic protein; GSK-3β: glycogen synthase kinase-3β; *Hdac2*: genes that encode histone deacetylase 2; Iba-1: ionized calcium binding adaptor molecule 1; IL-1β: interleukin 1β; IL-6: interleukin-6; iNOS: inducible nitric oxide synthase; *Iqgap2*: genes that encode Ras GTPase-activating-like protein; IκB: inhibitor of nuclear factor kappa-light-chain-enhancer of activated B cells; IκBα: inhibitor of nuclear factor kappa-light-chain-enhancer of activated B cells alpha; JNK: c-Jun N-terminal kinase; MAPK: mitogen-activated protein kinase; MCP1: monocyte chemoattractant protein 1; Mdm2: mouse double minute 2 homolog; MEK: Mitogen-activated protein kinase kinase; mTOR: mammalian target of rapamycin; mRNA: messenger ribonucleic acid; NF-κB: nuclear factor kappa-light-chain-enhancer of activated B cells; NO: nitric oxide; PARP-1: poly (ADP-ribose) polymerase-1; p-GSK-3β: phosphorylated GSK-3β; PI3K: phosphoinositide 3-kinase; p-JNK: phosphorylated JNK; p-NF-κB: phosphorylated NF-κB; PKC: protein kinase C; PPARγ: peroxisome proliferator-activated receptor gamma; p70S6K: a ribosomal protein S6 kinase; RAGE: receptor for advanced glycation end products; SH-SY5Y: human-derived neuroblastoma cell line; SP600125: a specific JNK inhibitor; TLR4: Toll-like receptor 4; TNF-α: tumor necrosis factor α; 6-OHDA: 6-hydroxydopamine; ↑: increased; ↓: decreased.
